# Impact of inflammatory biomarkers and surgical interventions on one-month recovery after rib fractures: A propensity-matched cohort study

**DOI:** 10.1016/j.sopen.2025.10.009

**Published:** 2025-11-03

**Authors:** Xiaojiao Zhu, Jianwei Han, Chuan Long, Wenjun Cao, Suwei Xu, Yingding Ruan

**Affiliations:** a Department of Thoracic Surgery, The First People's Hospital of Jiande, Jiande, China; b Department of Thoracic Surgery, Affiliated Zhongshan Hospital of Dalian University, Dalian, 116044, China

**Keywords:** Rib fractures, Inflammatory biomarkers, Surgical intervention, Propensity score matching, Postoperative recovery

## Abstract

**Background:**

This study aims to assess the collective influence of inflammatory indicators and surgical interventions on the one-month postoperative recovery outcomes in patients with rib fractures.

**Methods:**

A retrospective analysis involved 70 surgical and 278 non - surgical rib - fracture patients. Primary outcomes were thoracic complication incidence and post - discharge oral analgesic use at one month. Secondary outcomes included hospital stay length and total costs. We collected various data and conducted propensity score matching (1:2 ratio) to control for confounders, followed by multivariate analyses.

**Results:**

After PSM (60 surgical vs. 117 non-surgical patients), surgical reduced hospital stay by 10.4 days (β = −10.36 days, 95 % confidence interval [95 % CI]: −16.03 to −4.70; *P* < 0.001), but increase in total costs by 30,808.80 CNY (P < 0.001). Pre-existing thoracic complications independently predicted higher one-month postoperative complications (OR[95 % CI] = 4.05 [1.08, 18.15]; *P* = 0.048), while comorbidities lowered risk (OR[95 %CI] = 0.29 [0.08, 0.89]; *P* = 0.043). Elevated systemic immune-inflammation index (SII) (Coef [95 % CI] = 528.03 [28.05, 1028.00]; *P* = 0.039) and neutrophil-to-lymphocyte ratio (NLR) (Coef[95 % CI] = 3.50 [0.62, 6.37]; *P* = 0.017) were positively correlated with Injury Severity Score (ISS). In surgical patients, a higher lymphocyte-to-monocyte ratio (LMR) independently predicted a lower likelihood of ongoing analgesic use at one month (OR[95 %CI] = 0.70 [0.46, 0.95]; *P* = 0.046).

**Conclusion:**

Surgical rib - fracture stabilization shortens hospital stay but raises treatment costs. High SII and NLR, along with thoracic complications, are linked to post - op complications. LMR and HGB levels are associated with analgesic needs, which may aid in tailored pain management.

## Graphical abstract

**Figure:**
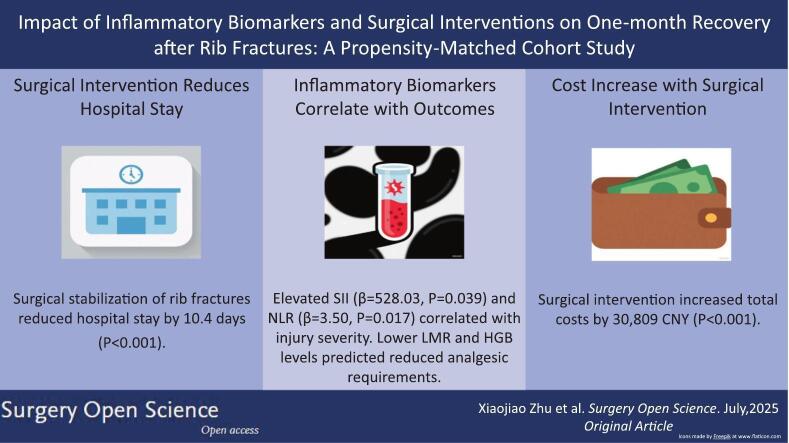

## Introduction

Thoracic trauma continues to be a major contributor to global mortality, ranking as the third leading cause of trauma-related death worldwide and frequently serves as a critical factor in secondary causes of mortality [1]. Annually, an estimated 1.98 million individuals globally experience rib fractures [2]. The treatment of rib fractures is a critical aspect of surgical care, particularly in patients with pre-existing conditions such as respiratory infections or cardiovascular diseases. These comorbidities significantly influence recovery pathways and frequently complicate treatment strategies [[3], [4], [5]].

The management of rib fractures requires a comprehensive approach that accounts for surgical indications, pain control strategies, and the patient's overall health status [3,[5], [6], [7], [8], [9], [10]]. Recent research has increasingly focused on the potential of inflammatory biomarkers as prognostic indicators in trauma patients. Biomarkers, such as the systemic immune-inflammation index (SII = Platelet*Neutrophil/Lymphocyte), lymphocyte-to-monocyte ratio (LMR), platelet-to-lymphocyte ratio (PLR), and neutrophil-to-lymphocyte ratio (NLR), derived from routine hematological parameters, offer cost-effective and readily available indicators of inflammatory status and have exhibited prognostic value in trauma patients [[11], [12], [13], [14], [15]]. Elevated NLR and PLR have been associated with increased inflammatory activity and impaired immune function in various clinical settings, such as in patients with COVID-19, where higher NLR and PLR were linked to disease severity and complications [16]. Similarly, in cancer patients, elevated NLR and PLR have been shown to predict worse outcomes and may reflect a pro-inflammatory state that can delay recovery and increase complication rates [17].

Despite growing attention to inflammatory biomarkers, research on their prognostic value for rib fracture patients remains scarce. This study sought to investigate the combined influence of these inflammatory biomarkers and surgical intervention on one-month postoperative recovery outcomes in patients with rib fractures. By exploring these factors, we aim to contribute to the understanding of how systemic inflammatory responses might influence patient outcomes and potentially inform future management strategies.

## Patients and methods

### Study population and eligibility criteria

This retrospective evaluation included patients diagnosed with rib fractures at The First People's Hospital of Jiande (Jiande, China) from January 2020 to December 2023. The inclusion criterion was limited to patients with radiologically confirmed rib fractures. Exclusion criteria were as follows: (1) age under 18 years; (2) death within 24 h of admission; (3) transfer to other hospitals during hospitalization; (4) concurrent or consecutive surgical interventions unrelated to rib fractures; (5) incomplete clinical or imaging data; (6) hospital stay of fewer than 3 days post-admission; (7) presentation to the hospital more than 24 h after injury; (8) presence of only one or no discernible rib fractures on imaging; (9) pregnancy or being incarcerated. Blood samples were collected from all patients upon admission. Inflammatory markers analyzed included the NLR, PLR, LMR, and SII.

This study adhered to the principles outlined in the Declaration of Helsinki and received approval from the Ethics Committee of The First People's Hospital of Jiande (Ethics Committee Approval Number: 20250703-KY-002-01). Due to the retrospective nature of the research, informed consent was waived.

### Data collection

Clinical and demographic data were extracted from electronic medical records, including sex, age, body mass index (BMI), and smoking history. Comorbidities assessed included hypertension, diabetes mellitus, coronary heart disease, emphysema, and chronic obstructive pulmonary disease (COPD). Injury-related variables comprised the total number of rib fractures, number of displaced ribs, fracture laterality (unilateral or bilateral), presence of paraspinal rib fractures, Injury Severity Score (ISS), and thoracic complications following chest trauma (pneumothorax, subcutaneous emphysema, hemothorax, and pulmonary contusion). Treatment-related data included analgesic use one month post-injury, surgical approach, operative time, intraoperative blood loss, postoperative drainage time and volume, and total hospital stay. Laboratory variables such as serum albumin (ALB) and hemoglobin (HGB) levels on admission were also recorded. Additional variables included hospital cost, insurance or third-party payment status, and findings from one-month follow-up chest computed tomography (CT).

All patients with rib fractures underwent three-dimensional rib reconstruction and chest CT to further accurately characterize fracture morphology. A displaced fracture was defined when there is no cortical contact, either due to overlap of the ends of the rib fragments or distraction [18]. Paraspinal rib fractures were identified based on the distance of the fracture from the paraspinal line on imaging, defined as ≤10 mm from the paraspinal line. Analgesic requirements were evaluated by documenting parenteral analgesic administration for acute pain management within 7 days post-admission or post-surgery. During the 1-month postoperative follow-up, oral analgesics used were primarily Lofepramine/codeine, an opioid analgesic. Data collection and initial analysis were independently conducted by two investigators, with subsequent verification by a third author to ensure accuracy.

### Surgical procedure

**Fig. 1:**
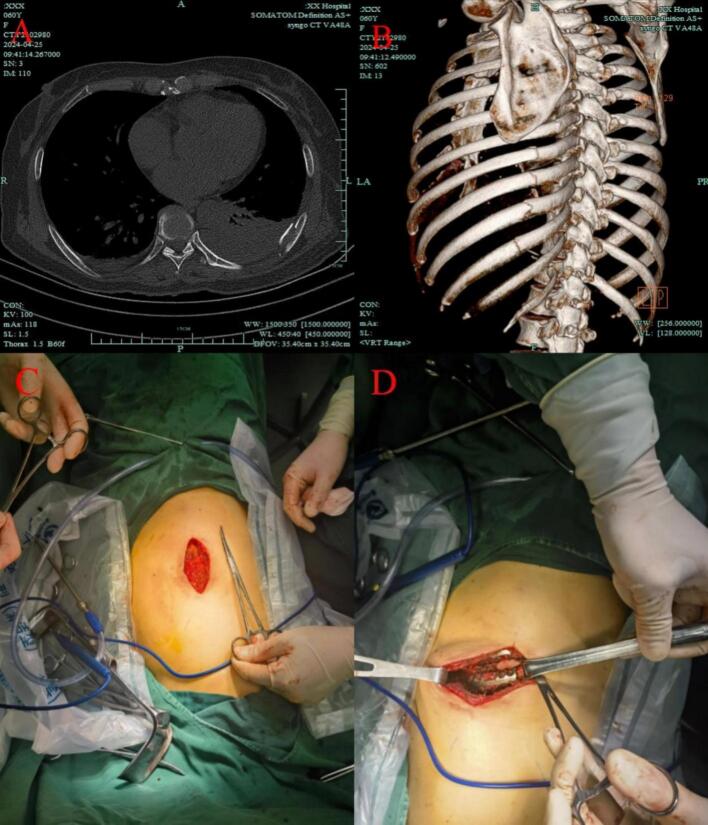
Surgical procedure. A: chest computed tomography; B: three - dimensional rib reconstruction; C: incision; D: titanium rib fixation plate.

All patients underwent preoperative chest CT and three - dimensional rib reconstruction (Fig. 1A–B). After general anesthesia with a single - lumen endotracheal tube, patients were placed in a supine or lateral position with the arm abducted. The surgical area was routinely disinfected and draped. The rib fracture site was located, and the skin was incised layer by layer (Fig. 1C). The subcutaneous tissue and muscles were dissected to expose the rib. A rib stripper was used to smooth the rib surface, extending approximately 2 cm beyond the fracture ends. The fractured rib ends were then reduced and secured with a titanium rib fixation plate (Fig. 1D). Hemostasis was achieved, residual blood in the pleural cavity was removed, and the cavity was irrigated. After confirming the completeness of the procedure, the incisions were closed layer by layer.

The titanium rib fixation plate used in this study is a contoured rib fixation plate, manufactured by HuaSeng Medical in Changzhou, China. This plate is specifically designed for rib fixation and is made of high - quality titanium alloy, offering excellent biocompatibility and mechanical strength.

### Observation indicators

This study investigated two categories of outcomes. The primary outcome comprised thoracic complications identified via chest CT one month post-injury, which included pulmonary infection, atelectasis, pleural effusion, pneumothorax, pain, and poor wound healing. The primary outcome also included the continued use of oral analgesics at the one - month follow - up. The secondary outcome included the length of hospital stay and total hospitalization costs. Follow-up chest CT scans were independently reviewed by two investigators (Jianwei Han and Xiaojiao Zhu). Complications at admission were recorded separately from those occurring post-injury to ensure accurate analysis of complication development and resolution.

### Statistical analysis

Propensity score matching (PSM) (1:2 ratio) was conducted to improve comparisons between surgical and non-surgical groups and reduce selection bias. Propensity scores were computed using a logistic regression model incorporating age, total number of rib fractures, number of displaced ribs, ISS, pre-admission HGB and ALB levels, and the presence of thoracic complications following chest trauma. We employed the nearest-neighbor matching technique with a caliper width of 0.2, without replacement, to ensure balanced potential confounder distribution between the groups (*P* > 0.05).

To assess covariate balance post-matching, we calculated the standardized mean difference (SMD). Generally, SMD values below 0.10 indicate good balance, 0.10–0.34 suggest minor imbalance, 0.35–0.64 indicate moderate imbalance, 0.65–1.19 reflect substantial imbalance, and values of 1.20 or higher signify a very large imbalance. Our analysis revealed that all covariates fell within the acceptable balance range. The SMD values are detailed in Table 1.

**Table 1:** Patient characteristics, rib fractures, and statistical analysis [n(%), mean ± standard deviation, M (P25, P75)].

Variables	Total (*n* = 348)	Before PSM			Total (*n* = 177)	After PSM			SMD
		Surgery(*n* = 70)	Non-Surgery (*n* = 278)	P-Value		Surgery(*n* = 60)	Non-Surgery (*n* = 117)	*p*-Value	
Sex, n(%)				0.063				0.404	0.135
Male	247 (71.0)	56 (80.0)	191 (68.7)		135 (76.3)	48 (80.0)	87 (74.4)		
Female	101 (29.0)	14 (20.0)	87 (31.3)		42 (23.7)	12 (20.0)	30 (25.6)		
Smoking, n(%)	156 (44.8)	29 (41.4)	127 (45.7)	0.522	80 (45.2)	24 (40.0)	56 (47.9)	0.320	0.159
Age(Mean ± SD)	59.67 ± 11.39	58.11 ± 9.84	60.06 ± 11.73	0.201	58.53 ± 11.09	57.88 ± 9.75	58.85 ± 11.74	0.583	0.091
BMI(Mean ± SD)	23.29 ± 3.16	23.43 ± 2.94	23.26 ± 3.21	0.688	23.17 ± 3.03	23.46 ± 2.65	23.03 ± 3.21	0.372	0.147
Comorbidities, n(%)	117 (33.6)	24 (34.29)	93 (33.45)	0.895	62 (35.0)	22 (36.7)	40 (34.2)	0.744	0.052
The number of rib fractures [M(P25, P75)]	5.00 (3.00, 7.00)	6.00 (4.00, 9.00)	4.00 (3.00, 6.00)	<0.001	5.00 (4.00, 7.00)	6.00 (4.00, 8.00)	5.00 (4.00, 7.00)	0.346	0.076
Rib fracture dislocation number [M(P25, P75)]	4.00 (3.00, 6.00)	4.50 (2.25, 7.00)	4.00 (3.00, 5.00)	0.103	4.00 (3.00, 6.00)	4.00 (2.00, 7.00)	4.00 (3.00, 5.00)	0.459	0.183
Location, n(%)				0.921				0.340	0.154
Unilateral	270 (77.6)	54 (77.1)	216 (77.7)		134 (75.7)	48 (80.0)	86 (73.5)		
Bilateral	78 (22.4)	16 (22.9)	62 (22.3)		43 (24.3)	12 (20.0)	31 (26.5)		
Paraspinal rib fractures, n(%)	98 (28.2)	23 (32.9)	75 (27.0)	0.328	39 (22.0)	18 (30.0)	21 (18.0)	0.067	0.285
ISS				0.001				0.569	0.189
≤16	300 (86.2)	52 (74.3)	248 (89.2)		142 (80.2)	47 (78.3)	95 (81.2)		
>16	35 (10.1)	10 (14.3)	25 (9.0)		23 (13.0)	7(11.7)	16 (13.7)		
>25	13 (3.7)	8 (11.4)	5 (1.8)		12 (6.8)	6 (10.0)	6 (5.1)		
*Thoracic complications following chest trauma, n(%)				<0.001				0.489	0.129
No Complications	136 (39.1)	14 (20.0)	122 (43.9)		44 (24.9)	14 (23.3)	30 (25.6)		
1 Complications	114 (32.8)	13 (18.6)	101 (36.3)		40 (22.6)	12 (20.0)	28 (23.9)		
Multiple complications (≥2)	98 (28.1)	43 (61.4)	55 (19.8)		93 (52.5)	34 (56.7)	59 (50.5)		
Analgesic, n(%)	73 (21.0)	18 (25.7)	55 (19.8)	0.276	44 (24.9)	16 (26.7)	28 (23.9)	0.690	0.063
Payment method, n(%)				0.790				0.502	0.1073
Insured	109 (31.3)	21 (30.0)	88 (31.6)		62 (35.0)	19 (31.7)	43 (36.8)		
Accident-related 3rd party claim	239 (68.7)	49 (70.0)	190 (68.4)		115 (65)	41 (68.3)	74 (63.2)		
Cost [M(P25, P75)]	9664.77 (6696.66, 20288.56)	45,691.16 (34,960.46, 59558.83)	8486.58 (6207.76, 11966.65)	<0.001	14,496.64 (9107.91, 35072.09)	45,542.18 (34,537.74, 55573.71)	10,021.31 (7707.34, 14496.64)	<0.001	2.086
Chest complications one month after injury, n(%)	75 (21.6)	13 (18.6)	62 (22.3)	0.497	33 (18.6)	12 (20.0)	21 (18.0)	0.740	0.052
Oral analgesic use at one-month follow-up, n(%)	64 (18.4)	14 (20.0)	50 (18.0)	0.697	26 (14.7)	12 (20.0)	14 (12.0)	0.153	0.221
ALB (Mean ± SD)	42.07 ± 4.26	41.15 ± 4.94	42.31 ± 4.04	0.042	41.05 ± 4.40	41.46 ± 5.02	40.84 ± 4.04	0.377	0.137
HGB(Mean ± SD)	129.39 ± 18.82	120.53 ± 19.22	131.62 ± 18.08	<0.001	122.36 ± 17.41	124.28 ± 16.98	121.37 ± 17.62	0.293	0.170
SII [M(P25, P75)]	701.21 (423.78, 1581.00)	798.58 (431.44, 1597.98)	698.35 (420.85, 1557.75)	0.685	790.00 (417.07, 1795.50)	741.00 (403.93, 1582.57)	793.75 (417.07, 1810.50)	0.909	0.062
LMR [M(P25, P75)]	3.25 (2.00, 5.00)	3.00 (1.76, 5.19)	3.33 (2.00, 5.00)	0.710	3.00 (1.80, 4.75)	3.00 (1.78, 4.90)	3.00 (1.80, 4.50)	0.677	0.082
PLR [M(P25, P75)]	123.73 (87.90, 183.44)	126.19 (79.30, 197.58)	123.14 (91.63, 183.12)	0.700	134.17 (84.74, 205.00)	131.67 (78.00, 195.60)	144.62 (88.42, 218.00)	0.253	0.030
NLR [M(P25, P75)]	4.10 (2.44, 8.55)	4.35 (2.75, 8.69)	4.00 (2.32, 8.46)	0.328	4.33 (2.50, 9.43)	4.18 (2.68, 8.81)	4.42 (2.46, 9.50)	0.920	0.037
Hospital stay[M(P25, P75)]	12.00 (7.00, 19.00)	10.00 (7.00, 16.75)	19.00 (13.00, 31.50)	<0.001	13.00 (9.00, 23.00)	19.00 (12.00, 30.50)	11.00 (8.00, 19.00)	<0.001	0.553

ALB, Albumin; BMI, Body Mass Index; CI, Confidence Interval; HGB, Hemoglobin; ISS, Injury Severity Score; SII, Preoperative Systemic Immune Inflammation Indices; LMR, Lymphocyte-to-Monocyte Ratio; M(P25,P75), Median(25th percentile,75th percentile); NLR, Meutrophil-to-Lymphocyte Ratio; PLR, Platelet-to-Lymphocyte Ratio; PMS, Propensity Score Matching; SD, Standard Deviation; S.E, Standard Error.

Normally distributed continuous variables were analyzed using Student's *t*-test and presented as means ± standard deviations. Non-normally distributed continuous variables were evaluated using the Wilcoxon rank-sum test and expressed as medians with interquartile ranges (25th–75th percentiles). Categorical variables underwent comparison using the Chi-square test or Fisher's exact test and were presented as percentages.

Univariate and multivariate analyses were conducted using binary logistic regression models. Variables with a *P*-value <0.05 in the univariate analysis were deemed significant and subsequently included in the multivariate model to identify independent predictors. The multivariate analysis employed backward elimination with a significance level of 0.05 for retention in the model. All statistical tests were two-sided, and a *P*-value of less than 0.05 was considered statistically significant. Statistical analyses were performed using SPSS version 22.0, ensuring rigorous control of biases and providing comprehensive insights into the relationships between the study variables.

## Results

### Demographic and baseline characteristics

**Fig. 2:**
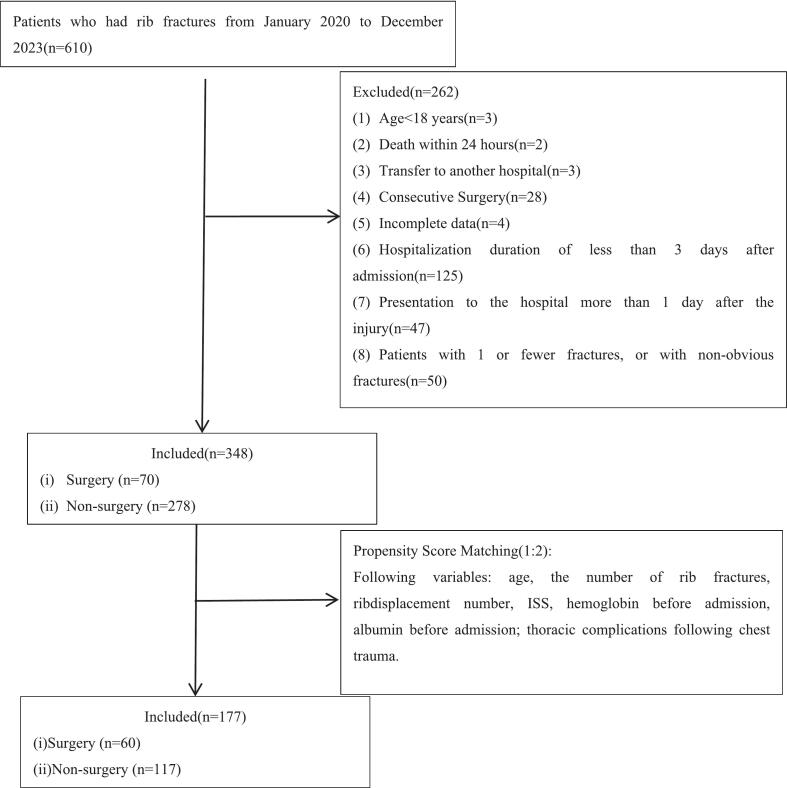
Flowchart of patient selection. ISS, injury severity score.

A total of 610 patients with radiologically confirmed rib fractures were screened for eligibility at The First People's Hospital of Jiande from January 2020 to December 2023. After applying the predefined inclusion and exclusion criteria, 348 patients were included in the study. Following PSM, 177 patients were included in the analysis, comprising 135 males (76.3 %) and 42 females (23.7 %), with a mean age of 58.53 ± 11.09 years. Of these, 60 patients (33.9 %) were in the surgical group and 117 (66.1 %) in the non-surgical group. The patient selection process is illustrated in Fig. 2, and a detailed comparison of baseline characteristics before and after matching is provided in Table 1.

PSM effectively eliminated confounding factors (Table 1). After matching, baseline characteristics were balanced between groups, except for variables inherently linked to injury severity. Prior to matching, the surgical group demonstrated significantly higher ISS (ISS >16: 14.3 % vs. 9.0 %; ISS >25: 11.4 % vs. 1.8 %, *P* = 0.001), hospitalization costs (median [P25, P75]: 45691.16 [34,960.46, 59558.83] vs. 8486.58 [6207.76, 11966.65], *P* < 0.001), number of rib fractures (median [P25, P75]: 6.00 [4.00, 9.00] vs. 4.00 [3.00, 6.00], P < 0.001), and incidence of thoracic complications following chest trauma (P < 0.001) compared to the non-surgical group. Admission ALB (surgical: 41.15 ± 4.94 vs. non-surgical: 42.31 ± 4.04 g/L, *P* = 0.042) and HGB levels (surgical: 120.53 ± 19.22 g/L vs. non-surgical: 131.62 ± 18.08 g/L, P < 0.001) also differed significantly. Following PSM, sex, age, BMI, comorbidities, smoking history, fracture laterality (unilateral vs. bilateral), ISS, number of rib fractures, thoracic complications following chest trauma, admission ALB and HGB levels, or payment method showed no significant intergroup differences (*P* > 0.05).

### Primary outcomes

The incidence of thoracic complications one month post-injury was assessed using binary unconditional logistic regression analysis (Table 2). The presence of comorbidities (odds ratio [OR]95 % confidence interval [95 %CI] = 0.29 [0.08, 0.89]; *P* = 0.043) was associated with an increased probability of complications one month post-injury. Furthermore, patients who experienced thoracic complications following chest trauma, including pneumothorax, subcutaneous emphysema, hemothorax, or pulmonary contusion (OR[95 %CI] = 4.05 [1.08, 18.15]; *P* = 0.048), were more likely to develop complications one month post-injury. Significantly, the occurrence of these complications one month post-injury was also associated with a subsequent need for continued analgesic use one month post-injury, suggesting a complex relationship between injury severity and postoperative pain management (OR[95 % CI] = 13.79 [5.14, 40.00]; *P* < 0.001).

**Table 2:** Results of univariate and multivariable logistic regression analyses for chest complications one month after injury.

Variables	Univariate logistic regression analyses					Multivariable logistic regression analyses				
	β	S.E	t	P	OR (95 % CI)	β	S.E	t	P	OR(95 % CI)
Group										
Non-surgery	Ref									
Surgery	0.13	0.40	0.33	0.740	1.14 (0.51, 2.50)					
Sex										
Female	Ref									
Male	0.41	0.49	0.83	0.409	1.50 (0.61, 4.28)					
Smoking										
No	Ref									
Yes	0.42	0.39	1.19	0.234	1.59 (0.74, 3.43)					
Age	−0.01	0.02	−0.27	0.789	1.00 (0.96, 1.03)					
BMI	−0.06	0.07	−0.90	0.370	0.94 (0.83, 1.07)					
Comorbidities										
No	Ref					Ref				
Yes	−1.59	0.56	−2.84	0.005	0.20 (0.06, 0.55)	−1.23	0.61	−2.02	0.043	0.29 (0.08, 0.89)
The number of rib fractures	−0.09	0.07	−1.32	0.187	0.91 (0.79, 1.04)					
Rib fracture dislocation number	0.08	0.07	1.28	0.200	1.09 (0.96, 1.23)					
Location										
Unilateral	Ref									
Bilateral	−0.21	0.47	−0.46	0.648	0.81 (0.30, 1.93)					
Paraspinal rib fractures										
Yes	Ref									
No	0.29	0.49	0.59	0.555	1.34 (0.54, 3.83)					
ISS										
≤16	Ref									
>16	−0.95	0.77	−1.23	0.218	0.39 (0.06, 1.43)					
>25	0.31	0.70	0.44	0.662	1.36 (0.29, 4.90)					
*Chest complications at accident										
No complications	Ref					Ref				
1 Complications	1.57	0.62	2.52	0.012	4.81 (1.52, 18.55)	1.40	0.71	1.98	0.048	4.05 (1.08, 18.15)
Multiple complications (≥2)	0.73	0.59	1.24	0.217	2.08 (0.71, 7.62)	0.83	0.66	1.25	0.211	2.29 (0.68, 9.55)
Analgesic										
No	Ref									
Yes	−0.73	0.52	−1.41	0.159	0.48 (0.16, 1.24)					
Payment method										
Accident-related 3rd party claim	Ref									
Insured	−0.44	0.43	−1.03	0.303	0.64 (0.27, 1.44)					
Cost										
Oral analgesic use at one-month follow-up										
No	Ref					Ref				
Yes	2.77	0.49	5.65	<0.001	15.94 (6.27, 43.39)	2.62	0.52	5.06	<0.001	13.79 (5.14, 40.00)
ALB	−0.06	0.04	−1.30	0.208	0.95 (0.87, 1.03)					
HGB	0.00	0.01	0.34	0.737	1.00 (0.98, 1.03)					
SII	0.00	0.00	0.50	0.618	1.00 (1.00, 1.00)					
LMR	0.06	0.08	0.72	0.469	1.06 (0.90, 1.23)					
PLR	−0.00	0.00	−0.10	0.919	1.00 (1.00, 1.00)					
NLR	−0.01	0.03	−0.24	0.814	0.99 (0.93, 1.05)					
Hospital stay	0.02	0.01	1.34	0.180	1.02 (1.04, 1.34)					

ALB, Albumin; BMI, Body Mass Index; CI, Confidence Interval; HGB, Hemoglobin; ISS, Injury Severity Score; SII, Preoperative Systemic Immune Inflammation Indices; LMR, Lymphocyte-to-Monocyte Ratio; M(P25,P75), Median(25th percentile,75th percentile); NLR, Meutrophil-to-Lymphocyte Ratio; PLR, Platelet-to-Lymphocyte Ratio; PMS, Propensity Score Matching; SD, Standard Deviation; S.E, Standard Error.

The examination of factors linked to the continued use of oral analgesics one month post-injury revealed that the occurrence of complications during this period was a significant predictor (OR[95 % CI] = 14.95 [5.59, 43.40]; P < 0.001). This finding emphasizes the critical nature of addressing chest complications promptly to alleviate prolonged pain and the subsequent necessity for pain management. No other factors demonstrated statistical significance in predicting the need for oral analgesic use one month following injury (Table 3).

**Table 3:** Results of univariate and multivariable logistic regression analyses for oral analgesic one month after injury.

Total										
Variables	Univariate logistic regression analyses					Multivariable logistic regression analyses				
	β	S.E	t	P	OR (95 %CI)	β	S.E	t	P	OR (95 %CI)
Group										
Non-surgery	Ref									
Surgery	0.61	0.43	1.42	0.157	1.84 (0.78, 4.29)					
Sex										
Male	Ref									
Female	0.04	0.50	0.09	0.933	1.04 (0.41, 3.03)					
Smoking										
No	Ref									
Yes	0.59	0.43	1.37	0.170	1.80 (0.78, 4.28)					
Age	−0.01	0.02	−0.49	0.622	0.99 (0.95, 1.03)					
BMI	−0.06	0.07	−0.82	0.412	0.94 (0.81, 1.08)					
Comorbidities										
No	Ref					Ref				
Yes	−1.23	0.57	−2.17	0.030	0.29 (0.08, 0.81)	−0.36	0.65	−0.56	0.577	0.70 (0.18, 2.36)
The number of rib fractures	−0.05	0.07	−0.65	0.519	0.95 (0.82, 1.09)					
Rib fracture dislocation number	0.04	0.07	0.54	0.590	1.04 (0.90, 1.19)					
Location										
Unilateral	Ref									
Bilateral	0.16	0.48	0.34	0.735	1.18 (0.43, 2.92)					
Paraspinal rib fractures										
Yes	Ref									
No	0.51	0.58	0.88	0.380	1.66 (0.59, 5.96)					
ISS										
≤16	Ref									
>16	−0.15	0.66	−0.22	0.826	0.86 (0.19, 2.81)					
>25	0.14	0.81	0.18	0.861	1.15 (0.17, 4.78)					
*Chest complications at accident										
No complications	Ref									
1 Complications	0.82	0.61	1.35	0.178	2.26 (0.71, 8.02)					
Multiple complications (≥2)	0.14	0.57	0.26	0.799	1.16 (0.40, 3.84)					
Analgesic										
No	Ref									
Yes	−0.38	0.53	−0.72	0.474	0.68 (0.22, 1.81)					
Payment method										
Accident-related 3rd party claim	Ref					Ref				
Insured	−1.23	0.57	−2.17	0.03	0.29 (0.08, 0.81)	−1.20	0.64	−1.87	0.061	0.30 (0.08, 0.98)
Cost	0.00	0.00	1.92	0.055	1.00 (1.00, 1.00)					
Chest complications one month after injury										
No	Ref					Ref				
Yes	2.77	0.49	5.65	<0.001	15.94 (6.27, 43.39)	2.70	0.52	5.22	<0.001	14.95 (5.59, 43.40)
ALB	0.03	0.05	0.54	0.590	1.03 (0.93, 1.13)					
HGB	−0.00	0.01	−0.24	0.814	1.00 (0.97, 1.02)					
SII	0.00	0.00	0.73	0.468	1.00 (1.00, 1.00)					
LMR	−0.09	0.10	−0.93	0.351	0.91 (0.74, 1.09)					
PLR	−0.00	0.00	−0.73	0.753	1.00 (0.99, 1.00)					
NLR	0.01	0.03	0.36	0.721	1.01 (0.95, 1.07)					
Hospital stay	0.02	0.01	1.52	0.127	1.02 (0.99, 1.04)					
Surgery group*										
LMR	−0.35	0.17	−2.02	0.044	0.71 (0.48, 0.95)	−0.36	0.18	−1.99	0.046	0.70 (0.46, 0.95)
HGB	−0.04	0.02	−2.01	0.044	0.96 (0.92, 1.00)	−0.04	0.02	−2.05	0.041	0.96 (0.91, 1.00)

ALB, Albumin; BMI, Body Mass Index; HGB, Hemoglobin; ISS, Injury Severity Score; ICU, Intensive Care Unit; CI, Confidence Interval; SII, Preoperative Systemic Immune Inflammation Indices; LMR, Lymphocyte-to-Monocyte Ratio; NLR, Meutrophil-to-Lymphocyte Ratio; PLR, Platelet-to-Lymphocyte Ratio; S.E, Standard Error; *Chest complications at accident: including pneumothorax or subcutaneous emphysema, hemothorax, and pulmonary contusion; *Chest complications at accident: including pneumothorax or subcutaneous emphysema, hemothorax, and pulmonary contusion; The Surgery Group* included parameters such as rib displacement number, surgical duration, intraoperative blood volume, drainage time, and drainage volume. Three variables—rib displacement number, surgical duration, and intraoperative blood loss volume—showed no significant intergroup differences (P > 0.05).

### Secondary outcomes

A multivariate linear regression analysis was performed to identify factors influencing the length of hospital stay (Table 4). Surgical intervention was significantly associated with a shorter hospital stay compared to conservative treatment (Coef[95 % CI] = −10.36 [−16.03, −4.70]; *P* < 0.001). Male patients exhibited shorter hospital stays than female patients (Coef[95 % CI] = −4.85 [−8.88, −0.82]; *P* = 0.019). Patients with bilateral rib fractures experienced longer hospital stays compared to those with unilateral fractures (Coef[95 % CI] = 4.41 [0.46, 8.36]; *P* = 0.029). Additionally, medical insurance was also correlated with a shorter hospital stay (Coef[95 % CI] = −4.35 [−7.93, −0.77]; *P* = 0.018). Hospital stay was also positively correlated with total hospitalization costs (OR[95 % CI] = 0.00 [0.00, 0.00]; *P* < 0.001).

**Table 4:** Results of univariate and multivariable logistic regression analyses for hospital day.

Variables	Univariate logistic regression analyses					Multivariable logistic regression analyses				
	Coef	S.E	t	P	95 % CI	Coef	S.E	t	P	95 % CI
Group										
Non-surgery	Ref					Ref				
Surgery	8.54	2.28	3.74	<0.001	4.03–13.05	−10.36	2.87	−3.61	<0.001	−16.03 to −4.70
Sex										
Female	Ref									
Male	−6.98	2.59	−2.70	0.008	−12.09 to −1.87	−4.85	2.04	−2.37	0.019	−8.88 to −0.81
Smoking										
No	Ref									
Yes	−2.90	2.247	−1.293	0.198	−7.34 to 1.53					
Age	0.02	0.10	0.24	0.812	−0.18 to 0.22					
BMI	−0.37	0.37	−1.00	0.320	−1.10 to 0.36					
Comorbidities										
No	Ref									
Yes	−3.81	2.34	−1.63	0.105	−8.42 to 0.80					
The number of rib fractures	0.52	0.37	1.41	0.159	−0.21 to 1.25					
Rib fracture dislocation number	0.98	0.39	2.53	0.012	0.22–10.45	−0.26	0.33	−0.77	0.442	−0.92 to 0.40
Location										
Unilateral	Ref					Ref				
Bilateral	5.35	2.59	2.07	0.040	0.22–1.74	4.41	2.00	2.20	0.029	0.46–8.36
Paraspinal rib fractures										
Yes	Ref									
No	−4.98	2.68	−1.86	0.065	−10.28 to 0.32					
ISS										
≤16	Ref									
>16	2.43	3.34	0.73	0.468	−4.16 to 9.01					
>25	8.00	4.46	1.79	0.074	−0.80 to 16.81					
*Chest complications at accident										
No complications	Ref									
1 Complications	−4.24	3.23	−1.31	0.191	−10.62 to 2.14					
Multiple complications (≥2)	1.81	2.71	0.67	0.505	−3.54 to 7.15					
Analgesic										
No	Ref									
Yes	1.01	2.60	0.39	0.700	−4.12 to 6.14					
Payment method										
Accident-related 3rd party claim	Ref					Ref				
Insured	−6.49	2.30	−2.82	0.005	−4.12 to −1.95	−4.35	1.81	−2.40	0.018	−7.93 to −0.77
Cost	0.00	0.00	9.15	<0.001	0.00–0.00	0.00	0.00	8.39	<0.001	0.00–0.00
Chest complications one month after injury										
No	Ref									
Yes	3.91	2.87	1.36	0.174	−1.75 to 9.58					
Oral analgesic use at one-month follow-up										
No	Ref									
Yes	4.93	3.15	1.56	0.120	−1.29 to 0.21					
ALB	−0.29	0.26	−1.14	0.257	−1.29 to 11.15					
HGB	0.01	0.06	0.15	0.884	−0.12 to 0.14					
SII	0.00	0.00	0.52	0.605	0.00–0.00					
LMR	1.16	0.46	2.49	0.014	−0.24 to 2.07	0.56	0.36	1.54	0.125	−0.16 to 1.28
PLR	0.01	0.01	0.84	0.401	−0.01 to 0.03					
NLR	−0.02	0.17	−0.11	0.916	−0.36 to 0.32					
Surgery group*										
Sex										
Female	Ref									
Male	−15.23	5.94	−2.57	0.013	−27.11 to −3.34	−8.26	5.13	−1.61	0.114	−18.57 to 2.04
Rib fracture dislocation number										
Yes	Ref									
No	1.81	0.78	2.31	0.024	0.24–3.38	−0.12	0.83	−0.15	0.883	−1.79 to 1.55
ICU										
No	Ref					Ref				
Yes	17.35	7.75	2.24	0.029	1.84–32.87	2.43	7.77	0.31	0.756	−13.17 to 18.04
Drainage time	0.01	0.00	2.18	0.033	0.00–0.01	0.00	0.00	−1.34	0.187	−0.01 to 0.00
Drainage volume	3.42	0.84	4.07	<0.001	1.74–5.10	2.32	1.1	2.11	0.039	0.12–4.53
ALB	−0.98	0.47	−2.10	0.04	−1.91 to −0.05	−0.56	0.43	−1.30	0.200	−1.42 to 0.30
SII	0	0	−0.97	0.335	−0.01 to 0.00					
LMR	2.93	0.91	3.21	0.002	1.1–4.76	1.99	0.83	2.40	0.020	0.32–3.66

ALB, Albumin; BMI, Body Mass Index; HGB, Hemoglobin; ISS, Injury Severity Score; ICU, Intensive Care Unit; CI, Confidence Interval; SII, Preoperative Systemic Immune Inflammation Indices; LMR, Lymphocyte-to-Monocyte Ratio; NLR, Meutrophil-to-Lymphocyte Ratio; PLR, Platelet-to-Lymphocyte Ratio; S.E, Standard Error; *Chest complications at accident: including pneumothorax or subcutaneous emphysema, hemothorax, and pulmonary contusion; *Chest complications at accident: including pneumothorax or subcutaneous emphysema, hemothorax, and pulmonary contusion; The Surgery Group* included parameters such as rib displacement number, surgical duration, intraoperative blood volume, drainage time, and drainage volume. Three variables—rib displacement number, surgical duration, and intraoperative blood loss volume—showed no significant intergroup differences (P > 0.05).

Multivariate linear regression analysis further revealed that the number of displaced rib fractures and the length of hospital stay were significant predictors of total hospitalization costs (*P* < 0.05). Specifically, an increase in the number of displaced rib fractures was associated with higher total costs (Coef[95 % CI] = 1400.06 [613.27, 2186.85]; P < 0.001). Similarly, a longer hospital stay predicted higher total costs (Coef[95 % CI] = 539.39 [416.08, 662.70]; P < 0.001) (Table 5).

**Table 5:** Results of univariate and multivariable logistic regression analyses for cost.

Variables	Univariate logistic regression analyses					Multivariable logistic regression analyses				
	Coef	S.E	t	P	95 % CI	Coef	S.E	t	P	95 % CI
Group										
Non-surgery	Ref					Ref				
Surgery	36,819.53	2416.70	15.24	<0.001	32,049.91–41,589.15	30,808.80	1940.25	15.88	<0.001	26,978.39–34,639.21
Sex										
Female	Ref									
Male	−1956.97	4098.82	−0.48	0.634	−10,046.45 to 6132.50					
Smoking										
No	Ref									
Yes	−3128.68	3497.93	−0.89	0.372	−10,032.24 to 3774.89					
Comorbidities										
No	Ref									
Yes	1627.46	3655.46	0.45	0.657	−5587.00 to 8841.93					
The number of rib fractures	1293.48	569.18	2.27	0.024	170.15–2416.82	9.91	350.63	0.03	0.978	−682.29 to 702.12
Rib fracture dislocation number	2668.65	577.10	4.62	<0.001	1529.67–3807.63	1400.06	398.54	3.51	<0.001	613.27–2186.85
Paraspinal rib fractures										
No	Ref					Ref				
Yes	−14,601.95	4062.51	−3.59	<0.001	−22,619.77 to −6584.13	−3242.84	2329.59	−1.39	0.166	−7841.88 to 1356.20
ISS										
≤16	Ref					Ref				
>16	6006.53	5126.77	1.17	0.243	−4112.12 to 16,125.18	2161.28	2794.15	0.77	0.440	−3354.90 to 7677.45
>25	17,389.93	6857.02	2.54	0.012	3856.30–30,923.56	2232.68	3703.78	0.60	0.547	−5079.25 to 9544.62
*Chest complications at accident										
No complications	Ref									
1 Complications	856.72	5029.20	0.17	0.865	−9069.37 to 10,782.81					
Multiple complications (≥2)	7270.00	4212.19	1.73	0.086	−1043.56 to 15,583.57					
Analgesic										
No	Ref									
Yes	3472.23	4028.66	0.86	0.390	−4478.79 to 11,423.25					
Payment type										
Accident-related 3rd party claim	Ref									
Insured	−3576.90	3647.52	−0.98	0.328	−10,775.70 to 3621.90					
Age	135.22	157.49	0.86	0.392	−175.61 to 446.05					
BMI	−136.43	577.77	−0.24	0.814	−1276.72 to 1003.86					
Chest complications one month after surgery										
No	Ref									
Yes	6030.51	4456.92	1.35	0.178	−2765.72 to 14,826.74					
Oral analgesic use at one-month follow-up										
No	Ref					Ref				
Yes	9865.99	4872.24	2.03	0.044	250.08–19,481.90	2594.87	2514.84	1.03	0.304	−2369.90 to 7559.64
Station										
Unilateral	Ref									
Bilateral	−504.68	4068.43	−0.12	0.901	−8534.19 to 7524.83					
SII	0.85	1.52	0.56	0.576	−2.14 to 3.85					
LMR	834.43	730.62	1.14	0.255	−607.54 to 2276.40					
PLR	−13.68	18.22	−0.75	0.454	−49.63 to 22.28					
NLR	97.13	265.05	0.37	0.715	−425.98 to 620.24					
ALB	−503.93	396.30	−1.27	0.205	−1286.07 to 278.21					
HGB	−27.59	100.48	−0.28	0.784	−225.89 to 170.72					
Hospital day	883.82	96.55	9.15	<0.001	693.28–1074.37	539.39	62.46	8.64	<0.001	416.08–662.70
Surgery group*										
The number of rib fractures	2952.53	1043.96	2.89	0.006	862.82–5042.24	−511.77	977.20	−0.52	0.603	−2476.57 to 1453.03
Rib fracture dislocation number	4444.93	877.81	5.06	<0.001	2687.82–6202.05	−563.11	1383.27	−0.41	0.686	−3344.37 to 2218.14
ICU										
No	Ref					Ref				
Yes	34,839.92	9354.80	3.72	<0.001	16,114.25–53,565.59	11,879.37	8929.71	1.33	0.190	−6075.01 to 29,833.75
Number of fixed rib fractures	6922.93	1487.97	4.65	<0.001	3944.43–9901.42	4739.4	2044.16	2.32	0.025	629.35–8849.45
Antibiotics										
No	Ref					Ref				
Yes	17,485.38	8442.43	2.07	0.043	586.01–34,384.75	3799.18	6350.12	0.60	0.552	−8968.60 to 16,566.96
Drainage time	13.61	3.62	3.76	<0.001	6.36–20.86	0.65	4.12	0.16	0.876	−7.64 to 8.93
Drainage volume	4835.49	1049.24	4.61	<0.001	2735.20–6935.78	719.58	1303.46	0.55	0.584	−1901.21 to 3340.36
Operative time	194.78	45.56	4.28	<0.001	103.59–285.98	64.42	60.85	1.06	0.295	−59.72 to 186.76
ALB	−1445.17	593.93	−2.43	0.018	−2634.05 to −256.29	−487.52	503.50	−0.97	0.338	−1499.86 to 524.83
Hospital day	731.79	139.34	5.25	<0.001	452.87–1010.70	576.34	133.12	4.33	<0.001	308.69–844.00
Adjacent to the spine										
No	Ref					Ref				
Yes	−15,424.19	6403.16	−2.41	0.019	−28,241.50 to −2606.88	−4090.46	6070.58	−0.67	0.504	−16,296.18 to 8115.25

ALB, Albumin; BMI, Body Mass Index; HGB, Hemoglobin; ISS, Injury Severity Score; ICU, Intensive Care Unit; CI, Confidence Interval; SII, Preoperative Systemic Immune Inflammation Indices; LMR, Lymphocyte-to-Monocyte Ratio; NLR, Meutrophil-to-Lymphocyte Ratio; PLR, Platelet-to-Lymphocyte Ratio; S.E, Standard Error; *Chest complications at accident: including pneumothorax or subcutaneous emphysema, hemothorax, and pulmonary contusion; *Chest complications at accident: including pneumothorax or subcutaneous emphysema, hemothorax, and pulmonary contusion; The Surgery Group* included parameters such as rib displacement number, surgical duration, intraoperative blood volume, drainage time, and drainage volume. Three variables—rib displacement number, surgical duration, and intraoperative blood loss volume—showed no significant intergroup differences (P > 0.05).

### Subgroup analysis of rib fracture surgery patients

Among the 60 propensity-matched patients who underwent surgical intervention for rib fractures, a detailed subgroup analysis was performed to evaluate the influence of inflammatory biomarkers and surgical interventions on postoperative recovery outcomes.

### Primary endpoints

Logistic regression analysis revealed no statistically significant predictors of thoracic complications occurring within the first postoperative month among all covariates examined (*P* > 0.05) (Supplementary Table 1).

Binary unconditional logistic regression analysis revealed two admission biomarkers were significantly associated with the need for continued oral analgesic use one month post-injury. A lower LMR on admission was associated with a decreased likelihood of analgesic use (OR[95 % CI] = 0.70 [0.46, 0.95]; *P* = 0.046). Additionally, lower HGB levels on admission predicted a reduced probability of oral analgesic use one month post-injury (OR [95 % CI] = 0.96 [0.91, 1.00]; *P* = 0.041) (Table 3).

### Secondary endpoints

Multivariate linear regression analysis revealed several factors significantly associated with the length of postoperative hospital stay (Table 4). Postoperative drainage time emerged as a significant predictor, with an increase in drainage time correlating to a longer hospital stay (Coef[95 % CI] = 2.32 [0.12, 4.53]; *P* = 0.039). Furthermore, total cost (Coef[95 % CI] = 0.00 [0.00, 0.00]; *P* = 0.009) and a higher LMR upon admission were also predictive of extended hospital stays (Coef[95 % CI] = 1.99 [0.32, 3.66]; *P* = 0.02).

Multivariate linear regression analysis further revealed that the number of surgically treated displaced ribs used during surgery and the length of hospital stay were significant predictors of total hospital costs (*P* < 0.05). Specifically, Each additional displaced rib managed intraoperatively was associated with higher total costs (Coef[95 % CI] = 4739.40 [629.35, 8849.45]; *P* = 0.025). Similarly, a longer hospital stay predicted higher total costs (Coef[95 % CI] = 576.34 [308.69, 844.00]; *P* < 0.001) (Table 5).

### Additional inflammatory biomarker analyses

Further analyses explored the relationships between inflammatory biomarkers and other variables. A trend was observed between the SII and ISS, although the difference between SII and ISS scores ≤16 and > 16 was not statistically significant (*P* = 0.344). However, among patients with ISS scores >25, a negative coefficient suggested a potential decrease in a related variable (Coef[95 % CI] = −523.05 [−1045.14, −0.96]; *P* = 0.050) (Supplementary Table 2). Additionally, the NLR upon admission was significantly associated with an increase in a related variable, indicating a strong relationship between NLR and postoperative outcomes (Coef[95 % CI] = 158.43 [127.58, 189.29]; *P* < 0.001) (Supplementary Table 3).

Furthermore, a significant inverse relationship was observed between LMR and PLR upon admission, as evidenced by a negative coefficient (Coef[95 % CI] = −0.01 [−0.01, −0.00]; *P* = 0.005). The length of hospital stay also demonstrated predictive value for changes in admission LMR, suggesting that extended hospitalization may be associated with alterations in inflammatory biomarker levels (OR[95 % CI] = 0.05 [0.02, 0.07]; *P* = 0.003) (Supplementary Table 4).

Lastly, significant associations were observed between NLR upon admission and SII (Coef[95 % CI] = 0.00 [0.00, 0.01]; *P* < 0.001), as well as between LMR upon admission and NLR (OR[95 % CI] = −0.42 [−0.82, −0.03];*P* = 0.037). These findings further underscore the complex interrelationship among various inflammatory biomarkers in patients with rib fractures (Supplementary Table 3).

Notably, PLR upon admission was also found to be predictive of postoperative complications, with a negative coefficient suggesting a potential protective effect against complications (Coef [95 % CI] = −57.92 [−115.67, −0.17]; *P* = 0.049). However, this finding should be interpreted with caution due to the narrow confidence interval, highlighting the need for further validation in larger cohorts (Supplementary Table 5).

## Discussion

This propensity score-matched study demonstrated that surgical stabilization of rib fractures significantly reduced the length of hospital stay by 10.4 days (*P* < 0.001), albeit with an increased total cost of 30,809 Chinese Yen (CNY) (P < 0.001). These findings align with previous research indicating that surgical intervention expedites recovery while incurring a higher economic burden [3,4]. Thoracic injuries were identified as a key predictor of postoperative complications (OR = 4.05, *P* = 0.048). Interestingly, the presence of comorbidities was associated with a reduced risk of complications (OR = 0.29, *P* = 0.043), potentially due to enhanced monitoring and care for these patients.

The elevated SII and NLR observed in our study are thought to reflect a pro-inflammatory state that may prolong systemic inflammation, delay healing processes, and increase the risk of postoperative complications. For example, studies have shown that elevated NLR and PLR are associated with increased levels of pro-inflammatory cytokines and impaired immune function, which can lead to delayed recovery and higher complication rates in various clinical conditions [16,17]. Conversely, a higher LMR may indicate a more balanced immune response, potentially contributing to better pain management and recovery. These biomarkers offer a snapshot of the patient's inflammatory status, which can guide clinical decisions and improve patient outcomes.

Elevated inflammatory biomarkers, specifically preoperative systemic immune inflammation indices (SII) (β = 528.03, *P* = 0.039) and NLR (β = 3.50, *P* = 0.017), demonstrated a correlation with injury severity, suggesting their potential utility in identifying high-risk patients. The predictive value of these biomarkers may be attributed to their ability to reflect dynamic immune alterations following trauma. Notably, male patients exhibited shorter hospital stays (β = −4.85 days, *P* = 0.019), a finding that may be attributed to socioeconomic or physiological factors, warranting further investigation.

Changes in inflammatory biomarkers among rib fracture patients highlight the complex interplay between systemic inflammatory responses and clinical outcomes, necessitating interpretation within specific injury profiles. This study demonstrates that lower admission LMR and HGB levels independently predict reduced analgesic requirements. This finding suggests immuno-hematological regulatory mechanisms within pain pathways, where lymphopenia may disrupt endogenous pain modulation, while monocyte-driven inflammation amplifies nociceptive signaling [19,20]. The observed association between lower HGB levels and reduced analgesic requirements is intriguing but not fully explained by our findings. Future research should investigate the physiological basis for this relationship to better understand its clinical implications.

Additionally, LMR is closely associated with prognostic outcomes. In 2024, Li et al. [21] reported significantly lower LMR in the stroke-associated pneumonia (SAP) group compared to the non-SAP group (2.46 ± 1.44 vs. 3.86 ± 1.48, *P* < 0.001), revealing a nonlinear association between LMR and SAP incidence. Subgroup analysis demonstrated an inverse correlation between LMR <4 and SAP incidence, establishing LMR as an independent predictor of SAP (OR[95 % CI] = 0.37 [0.27–0.53]). In oncology, Wang et al. [22] found that elevated monocyte-to-lymphocyte ratios (MLR) in hepatocellular carcinoma patients significantly correlated with recurrence, early recurrence, and poorer overall survival. The 1-, 3-, and 5-year cumulative OS rates were substantially lower in the high-MLR group (P < 0.001). Conversely, Ruan et al. [13] observed no significant association between preoperative inflammatory markers, including LMR, and postoperative atrial fibrillation incidence in lung cancer patients.

Notably, PLR exhibited an inverse correlation with postoperative thoracic complications (Coef [95 % CI] = −57.92 [−115.67, −0.17]; *P* = 0.049), indicating its potential as a biomarker for predicting postoperative risks. This injury-specific immunologic shift, combined with dynamic platelet-lymphocyte interactions, emphasizes the need for mechanistic studies to elucidate the dual role of PLR across different trauma subtypes. For example, Rau et al. found no predictive value for NLR or PLR in a mixed-trauma cohort, which included head/neck injuries and resuscitation interventions [23]. In contrast, Melinte et al. [24] reported that elevated baseline hematologic ratios, including PLR, independently predicted deep vein thrombosis after total knee arthroplasty. Similarly, a previous study on periprosthetic joint infections revealed significant associations between NLR/PLR and early periprosthetic joint infections, with NLR demonstrating stronger predictive capacity (cut-off: 2.77; sensitivity: 84.6 %; specificity: 89.7 %; 95 % CI: 0.86–0.97) [25]. The inverse relationship between LMR and PLR, along with PLR's protective effect against complications, further underscores the complex, dynamic interplay among inflammatory mediators.

However, findings regarding the NLR have demonstrated variability across trauma populations. Soulaiman et al. [26] showed that elevated NLR on post-trauma day 1 strongly predicted 30-day survival, although this association was not independent of other covariates. A separate study examining inflammatory markers and mortality in severely injured patients found no significant difference in NLR between polytrauma non-survivors and survivors [23]. In contrast, another investigation revealed that NLR ≥ cut-off values correlated with increased inpatient mortality (*p* < 0.001, log-rank test), with NLR >8.19 and > 7.92 independently predicting mortality on days 2 and 5, respectively [27]. The clinical utility of inflammatory ratios depends not only on absolute values but also on interpreting dynamic fluctuations, as trauma may confound biomarker trajectories—suggesting that single measurements might underestimate the prognostic capacity of NLR and LMR.

Furthermore, this study employed multivariable linear regression analysis among rib fracture patients, revealing significant positive correlations between inflammatory markers and injury severity. Specifically, the SII demonstrated a significant association with ISS when ISS exceeded 16 (Coef [95 % CI] = 528.03 [28.05, 1028.00]; *P* = 0.039). Similarly, the NLR showed a significant relationship with ISS >16 (*P* = 0.017). Both SII and NLR were significantly associated with a composite metric of thoracic complications, encompassing pneumothorax, subcutaneous emphysema, hemothorax, and pulmonary contusion. The PLR exhibited a significant positive correlation with ISS when ISS surpassed 25 (Coef [95 % CI] = 56.55 [0.61, 112.49]; *P* = 0.048).

It should also be noted that while the Injury Severity Score (ISS) was used to adjust for injury severity in propensity matching, ISS may have limitations in fully capturing the complexity of injuries in patients with multiple trauma. We excluded patients with severe polytrauma requiring surgery for multiple body regions to help ensure the validity of our analysis. In patients undergoing surgical management for rib fractures, our analysis revealed specific associations between the SII, NLR, and clinical outcomes. Notably, SII approached borderline statistical significance at Injury Severity Score (ISS) >25 (Coef [95 % CI] = −523.05 [−1045.14, −0.96]; *P* = 0.050). While admission NLR levels did not directly correlate with ISS, they demonstrated significant interactions with other inflammatory markers.

These findings are consistent with previous research. A study investigating predictors of pneumothorax in thoracic trauma identified elevated admission levels of NLR, MLR, PLR, SII, systemic immune-inflammation response index (SIRI), and aggregate inflammatory systemic index (AISI) as highly predictive of pneumothorax development [28]. Similarly, another investigation demonstrated that preoperative elevations in hematologic markers such as MLR, NLR, PLR, SII, SIRI, and AISI strongly predicted the risk of acute deep vein thrombosis following total knee arthroplasty [24]. Our study not only corroborates these conclusions but also extends the understanding of the predictive utility of these inflammatory biomarkers across different ISS strata and specific complication profiles.

Consequently, future protocols should incorporate sequential biomarker evaluations to monitor immune recovery trajectories and inform personalized interventions. Furthermore, research should focus on refining biomarker thresholds and integrating inflammatory profiles with clinical factors, including surgical timing, comorbidities, and cultural context, to optimize rib fracture management through balanced biomechanical and immunologic approaches. These multifaceted strategies show potential for developing precision-based treatment protocols tailored to individual patients, ultimately improving clinical outcomes.

The efficacy of surgical stabilization of rib fractures (SSRF) in postoperative recovery remains a topic of ongoing debate, with conflicting evidence reported across various studies [8,[29], [30], [31]]. Over the past decade, the utilization rates of SSRF have increased significantly—from less than 1 % to over 14 %. While discussions continue regarding its clinical utility, SSRF has gained widespread acceptance for patients with flail chest and ventilator dependence [8,29,32].

Proponents of SSRF highlight its immediate benefits, including reduced mechanical ventilation duration, shorter intensive care unit (ICU) stays, and decreased pneumonia incidence [33]. Meta-analyses support these advantages, demonstrating reduced mortality and pulmonary infections following SSRF [7,29,34]. Notably, SSRF lowered mortality in non-intubated patients to 1.6 % compared to 4.8 % in controls—a reduction significantly exceeding the 5.2 % mortality rate predicted by the Revised Injury Severity Classification score [9]. However, contradictory outcomes have also been reported. Hoepelman et al. [30] observed increased postoperative hospital days, diminished quality of life at one year, and implant-related irritation in a substantial proportion of patients. Similarly, Marasco et al. [35,36] found no improvement in quality of life 6–24 months postoperatively, despite shorter ICU stays for severe flail chest cases. Our study contributes novel insights to this conflicting evidence, demonstrating a 10.4-day reduction in hospital stay post-SSRF, potentially attributable to enhanced chest stability enabling early mobilization. However, we found no significant benefits in primary outcomes such as postoperative chest complications or analgesic use at one month. Notably, neither the number of fixation plates nor ribs stabilized correlated with complications or analgesic requirements, suggesting surgical scope may not be a critical factor in short-term recovery. Furthermore, our subgroup analysis revealed no significant association between time from admission to surgery and hospital stay, costs, or one-month outcomes—contradicting studies advocating early intervention [37,38]. However, cultural factors in China, where patients often extend hospital stays despite meeting discharge criteria, may partially confound this metric.

In Asian populations, SSRF has shown substantial advantages, including a 5.23-day decrease in mechanical ventilation duration, a 4-day reduction in ICU stay, and a 6.54-day shorter hospital length of stay. Moreover, SSRF was linked to a 54 % lower pneumonia risk and a 56 % decrease in atelectasis occurrence [15]. These results are consistent with global trends but highlight the necessity of developing population-specific protocols for non-flail chest injuries, especially in regions where surgical thresholds or cultural practices may vary [32,39,40].

Systematic reviews and randomized controlled trials investigating the efficacy of SSRF in non-flail chest injuries have produced inconsistent findings. Studies conducted in Asia reported reductions in pain levels and hospital stay durations [15,41], while Western trials observed no significant differences in 3-month pain scores or quality-of-life metrics [42]. These disparities may be attributed to variations in surgical timing, fixation techniques, or crossover bias, underscoring the necessity for standardized protocols in future research.

Implant-related irritation affected 26.9 %–32.1 % of patients, primarily due to chest wall rigidity, foreign body sensation, and neuropathic pain [43,44]. Postoperative chronic pain arises from multiple factors, including hardware-related issues, intercostal neuropathy, and fibrotic scarring, as evidenced in comparative analyses of surgical versus conservative strategies [39]. While innovative implants such as intramedullary nails and biodegradable plates show potential for reducing irritation, their effectiveness requires further investigation [45,46,49].

In our propensity score-matched cohort, baseline demographic factors, including sex, age, smoking status, comorbidities, and BMI, demonstrated no significant correlation with postoperative analgesic use or complications at one month. The surgical and non-surgical groups exhibited comparable age (57.85 ± 9.78 vs. 58.53 ± 11.09 years) and BMI (23.38 ± 2.70 vs. 23.17 ± 3.03), consistent with evidence suggesting that rib fracture outcomes are more influenced by injury severity than baseline demographics [47]. However, conflicting data exist regarding the impact of smoking. While most studies associate smoking with poorer outcomes, such as higher pneumonia rates [48,49], Grigorian et al. reported reduced ventilator days and mortality in smokers, potentially attributable to large sample size (57,619 smokers) or unmeasured confounders [50]. Our study observed no smoking-related effects, which may be attributed to strict exclusion of comorbidities or variations in postoperative protocols.

This study, as a single-center retrospective analysis, presents several inherent limitations. Firstly, despite the use of propensity score matching, unmeasured confounding factors—such as surgeon experience, rehabilitation protocols, and socioeconomic factors, which often influence length of stay—may persist. Secondly, the measurement of inflammatory biomarkers only at admission limits our ability to assess dynamic changes during the recovery process. Thirdly, the exclusion of severely injured patients requiring emergent intervention may reduce the generalizability of findings to high-risk populations. Fourthly, patients with rib fractures combined with fractures in other areas not requiring surgery may influence the use of analgesic drugs. Fifthly, our study did not account for pre-injury use of opioids and other narcotics, nor preexisting mental health issues such as alcohol and drug use disorders, which could potentially confound the outcomes related to pain medication use. Lastly, the small sample size in the surgical subgroup precludes definitive conclusions regarding rare complications or optimal plating configurations.

Despite these limitations, our study makes substantial contributions to rib fracture management. The utilization of propensity score matching mitigated selection bias, ensuring comparable baseline characteristics between surgical and non-surgical cohorts. To the best of our knowledge, this research represents the first comprehensive evaluation of SII, NLR, LMR, and PLR in rib fracture patients, elucidating their distinct roles in predicting analgesia requirements, complications, and resource utilization. The integration of biomarker profiles with clinical metrics, such as surgical timing and fracture burden, establishes a novel framework for personalized decision-making. Moreover, our findings highlight the translational potential of inflammatory indices in trauma care.

In conclusion, while surgical management of rib fractures reduces hospital stays, it incurs higher costs. Inflammatory biomarkers (SII, NLR, and LMR) and injury severity emerge as critical prognostic determinants. Early intervention (within 7 days) and minimal plating strategies may accelerate recovery, while low LMR and HGB levels could inform personalized analgesic protocols. Further studies involving larger populations with long-term follow-up are necessary to elucidate additional patient benefits.

The following are the supplementary data related to this article.

Supplementary Table 1 Results of univariate and multivariable logistic regression analyses for chest complications one month after injury. Supplementary Table 1

Supplementary Table 2 Results of univariate and multivariable logistic regression analyses for SII. Supplementary Table 2

Supplementary Table 3 Results of univariate and multivariable logistic regression analyses for NLR. Supplementary Table 3

Supplementary Table 4 Results of univariate and multivariable logistic regression analyses for LMR. Supplementary Table 4

Supplementary Table 5 Results of univariate and multivariable logistic regression analyses for PLR. Supplementary Table 5

## Human ethics and consent to participate declarations

This study complied with the Declaration of Helsinki and was approved by the Ethics Committee of The First People's Hospital of Jiande (Ethics Committee Approval Number: 20250703-KY-002-01). The requirement for informed consent was waived because of the retrospective nature of the study.

## Clinical trial number

Not applicable.

## CRediT authorship contribution statement

**Xiaojiao Zhu:** Methodology, Investigation, Data curation, Conceptualization, Writing – review & editing, Writing – original draft. **Jianwei Han:** Methodology, Investigation, Data curation. **Chuan Long:** Investigation, Data curation. **Wenjun Cao:** Investigation, Data curation. **Suwei Xu:** Methodology, Investigation, Data curation. **Yingding Ruan:** Supervision, Conceptualization, Writing – review & editing.

## Consent for publication

All presentations of case reports have consent for publication.

## Funding

This research was supported by grants from the Jiande Municipal Science and Technology Bureau (Grant No. 2023SJZX07).

## Declaration of competing interest

The authors declare no competing interests.

## Data Availability

Any researchers interested in this study could contact Xiaojiao Zhu (E-mail:zhu89jiao@sina.com) to request the data.

## References

[1] Callcut R.A., Kornblith L.Z., Conroy A.S., Robles A.J., Meizoso J.P., Namias N. (2019). The why and how our trauma patients die: a prospective multicenter Western Trauma Association study. J Trauma Acute Care Surg.

[2] GBD 2019 Fracture Collaborators (2021). Global, regional, and national burden of bone fractures in 204 countries and territories, 1990–2019: a systematic analysis from the Global Burden of Disease Study 2019. Lancet Healthy Longev.

[3] Margiotta E., Wenger I.E., Henglein J., Kuo Y.H., Boland P., Martella N. (2024). Implementation of a modified pain, inspiration, cough protocol in patients with traumatic rib fractures. J Surg Res.

[4] Prins J.T.H., Van Lieshout E.M.M., Overtoom H.C.G., Tekin Y.S., Verhofstad M.H.J., Wijffels M.M.E. (2021). Long-term pulmonary function, thoracic pain, and quality of life in patients with one or more rib fractures. J Trauma Acute Care Surg.

[5] Kim J.S., Chien C.Y., Lewis M.R., Benjamin E.R., Demetriades D. (2025). Surgical rib fixation in patients with cardiopulmonary disease improves outcomes. Eur J Trauma Emerg Surg.

[6] Battle C., O’Neill M., Barnett J., Hutchings H., Uzzell B., Toghill H. (2025). Patient and clinician perceptions of blunt chest trauma management and recovery: a qualitative study. Disabil Rehabil.

[7] Liu X., Xiong K. (2019). Surgical management versus non-surgical management of rib fractures in chest trauma: a systematic review and meta-analysis. J Cardiothorac Surg.

[8] Craxford S., Owyang D., Marson B., Rowlins K., Coughlin T., Forward D. (2022). Surgical management of rib fractures after blunt trauma: a systematic review and meta-analysis of randomised controlled trials. Ann R Coll Surg Engl.

[9] Huelskamp M.D., Duesing H., Lefering R., Raschke M.J., Rosslenbroich S., TraumaRegister DGU (2025). Surgical stabilisation of rib fractures in non-ventilated patients: a retrospective propensity-matched analysis using the data from the trauma registry of the German Trauma Society (TraumaRegister DGUⓇ). Eur J Trauma Emerg Surg.

[10] Favre P.A., de Molliens L., Petit L., Biais M., Carrié C. (2021). May the neutrophil-to-lymphocyte ratio at admission predict the occurrence and the severity of ARDS after blunt chest trauma patients? A retrospective study. Am J Emerg Med.

[11] Diem S., Schmid S., Krapf M., Flatz L., Born D., Jochum W. (2017). Neutrophil-to-lymphocyte ratio (NLR) and platelet-to-lymphocyte ratio (PLR) as prognostic markers in patients with non-small cell lung cancer (NSCLC) treated with nivolumab. Lung Cancer.

[12] Zeng G., Li X., Li W., Wen Z., Wang S., Zheng S. (2023). A nomogram model based on the combination of the systemic immune-inflammation index, body mass index, and neutrophil/lymphocyte ratio to predict the risk of preoperative deep venous thrombosis in elderly patients with intertrochanteric femoral fracture: a retrospective cohort study. J Orthop Surg Res.

[13] Ruan Y., Han J., Yang A., Ding Q., Zhang T. (2024). Impact of preoperative inflammatory indices and postoperative pneumonia on postoperative atrial fibrillation in patients with non-small cell lung cancer: a retrospective study. BMC Pulm Med.

[14] Yao W., Wang W., Tang W., Lv Q., Ding W. (2023). Neutrophil-to-lymphocyte ratio (NLR), platelet-to-lymphocyte ratio (PLR), and systemic immune inflammation index (SII) to predict postoperative pneumonia in elderly hip fracture patients. J Orthop Surg Res.

[15] He W., Yang Y., Salonga R., Powell L., Greiffenstein P., Prins J.T.H. (2023). Surgical stabilization of multiple rib fractures in an Asian population: a systematic review and meta-analysis. J Thorac Dis..

[16] Mińko A., Turoń-Skrzypińska A., Rył A., Mańkowska K., Cymbaluk-Płoska A., Rotter I. (2024). The importance of the concentration of selected cytokines (IL-6, IL-10, IL-12, IL-15, TNF-α) and inflammatory markers (CRP, NLR, PLR, LMR, SII) in predicting the course of rehabilitation for patients after COVID-19 infection. Biomedicines.

[17] Serban D., Stoica P.L., Dascalu A.M., Bratu D.G., Cristea B.M., Alius C. (2023). The significance of preoperative neutrophil-to-lymphocyte ratio (NLR), platelet-to-lymphocyte ratio (PLR), and systemic inflammatory index (SII) in predicting severity and adverse outcomes in acute calculous cholecystitis. J Clin Med.

[18] Edwards J.G., Clarke P., Pieracci F.M., Bemelman M., Black E.A., Doben A. (2020). Taxonomy of multiple rib fractures: results of the chest wall injury society international consensus survey. J Trauma Acute Care Surg.

[19] Moro-García M.A., Mayo J.C., Sainz R.M., Alonso-Arias R. (2018). Influence of inflammation in the process of T lymphocyte differentiation: proliferative, metabolic, and oxidative changes. Front Immunol.

[20] Cheng H.R., Song J.Y., Zhang Y.N., Chen Y.B., Lin G.Q., Huang G.Q. (2020). High monocyte-to-lymphocyte ratio is associated with stroke-associated pneumonia. Front Neurol.

[21] Li X., Zhou X., Wang H., Ruan B., Song Z., Zhang G. (2024). Association between lymphocyte-to-monocyte ratio and stroke-associated pneumonia: a retrospective cohort study. PeerJ.

[22] Wang Q., Qiao W., Liu B., Li J., Yuan C., Long J. (2022). The monocyte to lymphocyte ratio not only at baseline but also at relapse predicts poor outcomes in patients with hepatocellular carcinoma receiving locoregional therapy. BMC Gastroenterol.

[23] Rau C.S., Wu S.C., Tsai C.H., Chou S.E., Su W.T., Hsu S.Y. (2022). Association of white blood cell subtypes and derived ratios with a mortality outcome in adult patients with Polytrauma. Healthcare (Basel).

[24] Melinte R.M., Arbănași E.M., Blesneac A., Zolog D.N., Kaller R., Mureșan A.V. (2022). Inflammatory biomarkers as prognostic factors of acute deep vein thrombosis following the total knee arthroplasty. Medicina (Kaunas).

[25] Zhao G., Chen J., Wang J., Wang S., Xia J., Wei Y. (2020). Predictive values of the postoperative neutrophil-to-lymphocyte ratio, platelet-to-lymphocyte ratio, and lymphocyte-to-monocyte ratio for the diagnosis of early periprosthetic joint infections: a preliminary study. J Orthop Surg Res.

[26] Soulaiman S.E., Dopa D., Raad A.T., Hasan W., Ibrahim N., Hasan A.Y. (2020). Cohort retrospective study: the neutrophil to lymphocyte ratio as an independent predictor of outcomes at the presentation of the multi-trauma patient. Int J Emerg Med.

[27] Dilektasli E., Inaba K., Haltmeier T., Wong M.D., Clark D., Benjamin E.R. (2016). The prognostic value of neutrophil-to-lymphocyte ratio on mortality in critically ill trauma patients. J Trauma Acute Care Surg.

[28] Vunvulea V., Melinte R.M., Brinzaniuc K., Suciu B.A., Ivănescu A.D., Hălmaciu I. (2023). Blood count-derived inflammatory markers correlate with lengthier hospital stay and are predictors of pneumothorax risk in thoracic trauma patients. Diagnostics (Basel).

[29] Beks R.B., Peek J., de Jong M.B., Wessem K.J.P., Öner C.F., Hietbrink F. (2019). Fixation of flail chest or multiple rib fractures: current evidence and how to proceed. A systematic review and meta-analysis. Eur J Trauma Emerg Surg.

[30] Hoepelman R.J., Beeres F.J.P., Beks R.B., Sweet A.A.R., Ijpma F.F., Lansink K.W.W. (2023). Non-operative vs. operative treatment for multiple rib fractures after blunt thoracic trauma: a multicenter prospective cohort study. Eur J Trauma Emerg Surg.

[31] Marasco S.F., Nguyen Khuong J., Fitzgerald M., Summerhayes R., Sekandarzad M.W., Varley V. (2023). Flail chest injury-changing management and outcomes. Eur J Trauma Emerg Surg.

[32] Sawyer E., Wullschleger M., Muller N., Muller M. (2022). Surgical rib fixation of multiple rib fractures and flail chest: a systematic review and meta-analysis. J Surg Res.

[33] Wu W.M., Yang Y., Gao Z.L., Zhao T.C., He W.W. (2015). Which is better to multiple rib fractures, surgical treatment or conservative treatment?. Int J Clin Exp Med.

[34] Liu T., Liu P., Chen J., Xie J., Yang F., Liao Y. (2019). A randomized controlled trial of surgical rib fixation in polytrauma patients with flail chest. J Surg Res.

[35] Marasco S.F., Martin K., Niggemeyer L., Summerhayes R., Fitzgerald M., Bailey M. (2019). Impact of rib fixation on quality of life after major trauma with multiple rib fractures. Injury.

[36] Marasco S.F., Davies A.R., Cooper J., Varma D., Bennett V., Nevill R. (2013). Prospective randomized controlled trial of operative rib fixation in traumatic flail chest. J Am Coll Surg.

[37] Towe C.W., Bachman K.C., Ho V.P., Pieracci F., Worrell S.G., Moorman M.L. (2024). Early repair of rib fractures is associated with superior length of stay and total hospital cost: a propensity matched analysis of the national inpatient sample. Medicina (Kaunas).

[38] Pieracci F.M., Coleman J., Ali-Osman F., Mangram A., Majercik S., White T.W. (2018). A multicenter evaluation of the optimal timing of surgical stabilization of rib fractures. J Trauma Acute Care Surg.

[39] Bauman Z.M., Visenio M., Patel M., Sprigman C., Raposo-Hadley A., Pieper C. (2022). Comparison of long-term outcomes from rib fractures for patients undergoing both operative and non-operative management: a survey analysis. Eur J Trauma Emerg Surg.

[40] Peek J., Kremo V., Beks R., van Veelen N., Leiser A., Link B.C. (2022). Long-term quality of life and functional outcome after rib fracture fixation. Eur J Trauma Emerg Surg.

[41] Pieracci F.M., Leasia K., Bauman Z., Eriksson E.A., Lottenberg L., Majercik S. (2020). A multicenter, prospective, controlled clinical trial of surgical stabilization of rib fractures in patients with severe, NONFLAIL fracture patterns (chest wall injury society NONFLAIL). J Trauma Acute Care Surg.

[42] Marasco S.F., Balogh Z.J., Wullschleger M.E., Hsu J., Patel B., Fitzgerald M. (2022). Rib fixation in non-ventilator-dependent chest wall injuries: a prospective randomized trial. J Trauma Acute Care Surg.

[43] Li Y., Jiang K., Zhao T., Guo X., Liu K., Zhao Y. (2023). If we should remove internal fixation devices for rib fractures?. J Cardiothorac Surg.

[44] Choi J., Kaghazchi A., Sun B., Woodward A., Forrester J.D. (2021). Systematic review and meta-analysis of hardware failure in surgical stabilization of rib fractures: who, what, when, where, and why?. J Surg Res.

[45] Song J., Murillo L.L., Yang K., Wang T., Li J., Li Y. (2022). Revisable and high-strength wheel-spun alginate/graphene oxide based fibrous rods towards a flexible and biodegradable rib internal fixation system. Int J Biol Macromol.

[46] Pacheco K.A. (2019). Allergy to surgical implants. Clin Rev Allergy Immunol.

[47] Shulzhenko N.O., Zens T.J., Beems M.V., Jung H.S., O’Rourke A.P., Liepert A.E. (2017). Number of rib fractures thresholds independently predict worse outcomes in older patients with blunt trauma. Surgery.

[48] Resnick S., Inaba K., Okoye O., Nosanov L., Grabo D., Benjamin E. (2014). Impact of smoking on trauma patients. Ulus Travma Acil Cerrahi Derg.

[49] Farris S.G., Zvolensky M.J., Beckham J.C., Vujanovic A.A., Schmidt N.B. (2014). Trauma exposure and cigarette smoking: the impact of negative affect and affect-regulatory smoking motives. J Addict Dis.

[50] Grigorian A., Lekawa M., Dolich M., Schubl S.D., Doben A.R., Kuza C.M. (2020). Smoking is associated with an improved short-term outcome in patients with rib fractures. Eur J Trauma Emerg Surg.

