# Supplementary material for: Impact of inflammatory biomarkers and surgical interventions on one-month recovery after rib fractures: A propensity-matched cohort study
Source: Surg Open Sci. 2025 Nov 3;28:49–62. doi: 10.1016/j.sopen.2025.10.009 (PMC12746880; doi:10.1016/j.sopen.2025.10.009)
Supplement: Supplementary Table 1 — Results of univariate and multivariable logistic regression analyses for chest complications one month after injury. [file mmc1.docx]

| Supply Table 1:Results of univariate and multivariable logistic regression analyses for chest complications one month after injury | | | | | | | | | | |
| --- | --- | --- | --- | --- | --- | --- | --- | --- | --- | --- |
| Variables | Univariate logistic regression analyses | | | | | Multivariable logistic regression analyses | | | | |
|  | β | S.E | t | P | OR (95%CI) | β | S.E | t | P | OR (95%CI) |
| Sex |  |  |  |  |  |  |  |  |  |  |
| Female | Ref |  |  |  |  |  |  |  |  |  |
| Male | 0.14 | 0.86 | 0.17 | 0.868 | 1.15 (0.25, 8.35) |  |  |  |  |  |
| Smoking |  |  |  |  |  |  |  |  |  |  |
| NO | Ref |  |  |  |  |  |  |  |  |  |
| YES | -0.36 | 0.68 | -0.53 | 0.599 | 0.70 (0.17, 2.55) |  |  |  |  |  |
| Comorbidities |  |  |  |  |  |  |  |  |  |  |
| NO | Ref |  |  |  |  |  |  |  |  |  |
| YES | -1.19 | 0.83 | -1.43 | 0.152 | 0.31 (0.04, 1.32) |  |  |  |  |  |
| The number of rib fractures | 0.02 | 0.11 | 0.18 | 0.854 | 1.02 (0.81，1.28) |  |  |  |  |  |
| Rib fracture dislocation number | 0.08 | 0.10 | 0.78 | 0.436 | 1.08 (0.88，1.33) |  |  |  |  |  |
| Paraspinal rib fractures |  |  |  |  |  |  |  |  |  |  |
| NO | Ref |  |  |  |  |  |  |  |  |  |
| YES | 0.41 | 0.73 | 0.553 | 0.581 | 1.50(0.38, 1.33) |  |  |  |  |  |
| ISS |  |  |  |  |  |  |  |  |  |  |
| ≤16 | Ref |  |  |  |  |  |  |  |  |  |
| ＞16 | -16.38 | 1495.30 | -0.01 | 0.991 | 0.00 (0.00 ~ Inf) |  |  |  |  |  |
| ＞25 | -0.42 | 1.15 | -0.37 | 0.712 | 0.65 (0.07, 6.21) |  |  |  |  |  |
| *Chest complications at acciden |  |  |  |  |  |  |  |  |  |  |
| No Complications | Ref |  |  |  |  |  |  |  |  |  |
| 1 Complications | 2.15 | 1.19 | 1.80 | 0.072 | 8.57 (1.09, 182.88) |  |  |  |  |  |
| Multiple Complications (≥2) | 0.91 | 1.13 | 0.80 | 0.422 | 2.48 (0.37, 49.54) |  |  |  |  |  |
| Analgesic |  |  |  |  |  |  |  |  |  |  |
| NO | Ref |  |  |  |  |  |  |  |  |  |
| YES | -0.11 | 0.74 | -0.15 | 0.884 | 0.90 (0.18, 3.57) |  |  |  |  |  |
| Payment method |  |  |  |  |  |  |  |  |  |  |
| Accident-related 3rd party claim | Ref |  |  |  |  |  |  |  |  |  |
| Insured | 0.19 | 0.69 | 0.28 | 0.778 | 1.21 (0.29, 4.55) |  |  |  |  |  |
| Cost | 0.00 | 0.00 | 1.10 | 0.270 | 1.00 (1.00, 1.00) |  |  |  |  |  |
| Age | 0.04 | 0.04 | 1.21 | 0.226 | 1.05 (0.98, 1.13) |  |  |  |  |  |
| BMI | -0.04 | 0.12 | -0.33 | 0.745 | 0.96 (0.75, 1.22) |  |  |  |  |  |
| ICU |  |  |  |  |  |  |  |  |  |  |
| NO | Ref |  |  |  |  |  |  |  |  |  |
| YES | 1.61 | 0.89 | 1.80 | 0.072 | 5.00 (0.81, 31.15) |  |  |  |  |  |
| Location |  |  |  |  |  |  |  |  |  |  |
| Unilateral | Ref |  |  |  |  |  |  |  |  |  |
| Bilateral | -1.18 | 1.10 | -1.08 | 0.281 | 0.31 (0.02, 1.85) |  |  |  |  |  |
| **Number of Fixed Rib Fractures** | 0.05 | 0.18 | 0.29 | 0.773 | 1.05 (0.73, 1.49) |  |  |  |  |  |
| Antibiotics |  |  |  |  |  |  |  |  |  |  |
| NO | Ref |  |  |  |  |  |  |  |  |  |
| YES | -0.16 | 0.88 | -0.18 | 0.857 | 0.85 (0.17, 6.31) |  |  |  |  |  |
| Intraoperative bleeding volume | 0.00 | 0.01 | 0.45 | 0.653 | 1.00 (0.99, 1.01) |  |  |  |  |  |
| Drainage volume | 0.00 | 0.00 | 1.18 | 0.237 | 1.00 (1.00, 1.00) |  |  |  |  |  |
| Drainage time | 0.14 | 0.11 | 1.21 | 0.226 | 1.15 (0.91, 1.45) |  |  |  |  |  |
| Operative time | 0.00 | 0.01 | 0.53 | 0.600 | 1.00(0.99, 1.01) |  |  |  |  |  |
| **Injury-to-Surgery Time** |  |  |  |  |  |  |  |  |  |  |
| 1≤ | Ref |  |  |  |  |  |  |  |  |  |
| ＜7 | -0.85 | 0.95 | -0.90 | 0.370 | 0.43 (0.07, 3.46) |  |  |  |  |  |
| ≥7 | 0.22 | 1.04 | 0.22 | 0.830 | 1.25 (0.17, 11.55) |  |  |  |  |  |
| **Postoperative Complications** |  |  |  |  |  |  |  |  |  |  |
| NO | Ref |  |  |  |  |  |  |  |  |  |
| YES | 0.64 | 0.71 | 0.91 | 0.365 | 1.90 (0.44, 7.45) |  |  |  |  |  |
| ALB | -0.10 | 0.07 | -1.45 | 0.147 | 0.91 (0.79，1.03) |  |  |  |  |  |
| SII | 0.00 | 0.00 | -0.14 | 0.892 | 1.00 (1.00，1.00) |  |  |  |  |  |
| LMR | 0.07 | 0.13 | 0.585 | 0.559 | 1.08 (0.83，1.38) |  |  |  |  |  |
| PLR | -0.00 | 0.00 | -0.83 | 0.409 | 1.00( 0.99，1.00) |  |  |  |  |  |
| NLR | -0.02 | 0.05 | -0.38 | 0.703 | 0.98( 0.87，1.07) |  |  |  |  |  |
| HGB | -0.00 | 0.02 | -0.10 | 0.918 | 1.00 (0.96，1.04) |  |  |  |  |  |
| Hospital day | 0.01 | 0.02 | 0.73 | 0.463 | 1.01(0.98, 1.04) |  |  |  |  |  |
| Paraspinal rib fractures |  |  |  |  |  |  |  |  |  |  |
| NO | Ref |  |  |  |  |  |  |  |  |  |
| YES | 0.41 | 0.73 | 0.55 | 0.581 | 1.50 (0.38, 7.46) |  |  |  |  |  |
| Oral analgesic use at one-month follow-up |  |  |  |  |  |  |  |  |  |  |
| NO | Ref |  |  |  |  |  |  |  |  |  |
| YES | 1.00 | 0.69 | 1.46 | 0.145 | 2.71 (0.68, 10.47) |  |  |  |  |  |
| ALB, Albumin; BMI, Body Mass Index; HGB, Hemoglobin; ISS, Injury Severity Score; ICU, Intensive Care Unit; CI, Confidence Interval; SII, Preoperative Systemic Immune Inflammation Indices; LMR, Lymphocyte-to-Monocyte Ratio; NLR, Meutrophil-to-Lymphocyte Ratio; PLR, Platelet-to-Lymphocyte Ratio; S.E, Standard Error; .*Chest complications at accident: including pneumothorax or subcutaneous emphysema, hemothorax, and pulmonary contusion; | | | | | | | | | | |
