# Supplementary material for: Impact of inflammatory biomarkers and surgical interventions on one-month recovery after rib fractures: A propensity-matched cohort study
Source: Surg Open Sci. 2025 Nov 3;28:49–62. doi: 10.1016/j.sopen.2025.10.009 (PMC12746880; doi:10.1016/j.sopen.2025.10.009)
Supplement: Supplementary Table 2 — Results of univariate and multivariable logistic regression analyses for SII. [file mmc2.docx]

| Supply Table 2:Results of univariate and multivariable logistic regression analyses for SII | | | | | | | | | | |
| --- | --- | --- | --- | --- | --- | --- | --- | --- | --- | --- |
| Variables | Univariate logistic regression analyses | | | | | Multivariable logistic regression analyses | | | | |
|  | Coef | S.E | t | P | 95% CI | Coef | S.E | t | P | 95% CI |
| Sex |  |  |  |  |  |  |  |  |  |  |
| Female | Ref |  |  |  |  |  |  |  |  |  |
| Male | 706.92 | 426.22 | 1.66 | 0.103 | -146.26 - 1560.10 |  |  |  |  |  |
| Smoking |  |  |  |  |  |  |  |  |  |  |
| NO | Ref |  |  |  |  |  |  |  |  |  |
| YES | -556.25 | 336.71 | -1.65 | 0.104 | -1230.24 - 117.75 |  |  |  |  |  |
| Comorbidities |  |  |  |  |  |  |  |  |  |  |
| NO | Ref |  |  |  |  | Ref |  |  |  |  |
| YES | 699.76 | 341.74 | 2.05 | 0.045 | 15.69 - 1383.83 | 244.12 | 176.16 | 1.38 | 0.169 | -104.80 - 593.03 |
| The number of rib fractures | -1.60 | 60.17 | -0.03 | 0.979 | -122.04 - 118.84 |  |  |  |  |  |
| Rib fracture dislocation number | 34.67 | 56.77 | 0.61 | 0.544 | -78.97 - 148.31 |  |  |  |  |  |
| Paraspinal rib fractures |  |  |  |  |  |  |  |  |  |  |
| NO | Ref |  |  |  |  |  |  |  |  |  |
| YES | 107.67 | 362.57 | 0.30 | 0.768 | -618.10 - 833.44 |  |  |  |  |  |
| ISS |  |  |  |  |  |  |  |  |  |  |
| ≤16 | Ref |  |  |  |  | Ref |  |  |  |  |
| ＞16 | 1164.75 | 511.26 | 2.28 | 0.026 | 140.97 - 2188.52 | 528.03 | 253.29 | 2.09 | 0.039 | 28.05 - 1028.00 |
| ＞25 | 123.57 | 340.80 | 0.36 | 0.717 | -549.06 - 796.20 | -63.42 | 338.22 | -0.19 | 0.852 | -731.04- 604.20 |
| *Chest complications at acciden |  |  |  |  |  |  |  |  |  |  |
| No Complications | Ref |  |  |  |  |  |  |  |  |  |
| 1 Complications | 36.09 | 242.64 | 0.73 | 0.882 | -442.80 - 514.99 | 19.66 | 243.49 | 0.08 | 0.936 | -460.97 - 500.29 |
| Multiple Complications (≥2) | 670.70 | 203.22 | 3.00 | 0.001 | 269.60 - 1071.79 | 573.85 | 209.73 | 2.74 | 0.007 | 159.84 - 987.85 |
| Analgesic |  |  |  |  |  |  |  |  |  |  |
| NO | Ref |  |  |  |  |  |  |  |  |  |
| YES | -206.75 | 380.72 | -0.54 | 0.589 | -968.84 - 555.35 |  |  |  |  |  |
| Payment type |  |  |  |  |  |  |  |  |  |  |
| Accident-related 3rd party claim | Ref |  |  |  |  |  |  |  |  |  |
| Insured | 874.67 | 349.96 | 2.50 | 0.015 | 174.14 - 1575.20 | 286.00 | 170.82 | 1.67 | 0.100 | -56.94 - 628.93 |
| Cost | -0.00 | 0.01 | -0.55 | 0.586 | -0.02 - 0.01 |  |  |  |  |  |
| Age | 8.37 | 7.83 | 1.07 | 0.285 | -7.06 - 23.84 |  |  |  |  |  |
| BMI | -9.65 | 63.05 | -0.15 | 0.879 | -135.85 - 116.55 |  |  |  |  |  |
| ICU |  |  |  |  |  |  |  |  |  |  |
| NO | Ref |  |  |  |  |  |  |  |  |  |
| YES | 931.83 | 549.16 | 1.70 | 0.095 | -167.44 - 2031.10 |  |  |  |  |  |
| Location |  |  |  |  |  |  |  |  |  |  |
| Unilateral | Ref |  |  |  |  |  |  |  |  |  |
| Bilateral | -365.78 | 419.23 | -0.87 | 0.387 | -1204.96 - 473.39 |  |  |  |  |  |
| Number of Fixed Rib Fractures | 56.49 | 93.82 | 0.60 | 0.549 | -131.31 - 244.30 |  |  |  |  |  |
| Antibiotics |  |  |  |  |  |  |  |  |  |  |
| NO | Ref |  |  |  |  |  |  |  |  |  |
| YES | 169.84 | 472.18 | 0.36 | 0.720 | -775.32 - 1115.01 |  |  |  |  |  |
| Intraoperative bleeding volume | 0.93 | 3.27 | 0.29 | 0.777 | -5.62 - 7.49 |  |  |  |  |  |
| Drainage time | 0.04 | 0.22 | 0.192 | 0.848 | -0.39 - 0.48 |  |  |  |  |  |
| Drainage volume | 58.26 | 65.82 | 0.88 | 0.380 | -73.49 - 190.01 |  |  |  |  |  |
| Operative time | -1.11 | 2.82 | -0.39 | 0.696 | -6.75 - 4.53 |  |  |  |  |  |
| Injury-to-Surgery Time |  |  |  |  |  |  |  |  |  |  |
| 1≤ | Ref |  |  |  |  |  |  |  |  |  |
| ＜7 | -431.05 | 536.32 | -0.80 | 0.425 | -1505.01 - 642.90 |  |  |  |  |  |
| ≥7 | -386.50 | 623.70 | -0.62 | 0.538 | -1635.45 - 862.44 |  |  |  |  |  |
| Postoperative Complications |  |  |  |  |  |  |  |  |  |  |
| NO | Ref |  |  |  |  |  |  |  |  |  |
| YES | -54.65 | 399.01 | -0.14 | 0.891 | -853.35 - 744.05 |  |  |  |  |  |
| ALB | -42.69 | 33.22 | -1.29 | 0.204 | -109.18 - 23.80 |  |  |  |  |  |
| LMR | -301.61 | 56.54 | -5.34 | <0.001 | -414.79 - -188.43 | 14.73 | 42.12 | 0.35 | 0.728 | -69.83 - 99.28 |
| PLR | 6.22 | 1.17 | 5.31 | <0.001 | 3.88 - 8.56 | 0.80 | 0.82 | 0.97 | 0.337 | -0.86 - 2.45 |
| NLR | 163.63 | 10.61 | 15.43 | <0.001 | 142.39 - 184.86 | 158.43 | 15.37 | 10.31 | <0.001 | 127.58 - 189.29 |
| HGB | -3.20 | 10.02 | -0.32 | 0.751 | -23.25 - 16.85 |  |  |  |  |  |
| Hospital day | -8.81 | 9.07 | -0.97 | 0.335 | -26.97 - 9.34 |  |  |  |  |  |
| Adjacent to the spine |  |  |  |  |  |  |  |  |  |  |
| NO | Ref |  |  |  |  |  |  |  |  |  |
| YES | 107.67 | 362.57 | 0.30 | 0.768 | -618.10 - 833.44 |  |  |  |  |  |
| ALB, Albumin; BMI, Body Mass Index; HGB, Hemoglobin; ISS, Injury Severity Score; ICU, Intensive Care Unit; CI, Confidence Interval; SII, Preoperative Systemic Immune Inflammation Indices; LMR, Lymphocyte-to-Monocyte Ratio; NLR, Meutrophil-to-Lymphocyte Ratio; PLR, Platelet-to-Lymphocyte Ratio; S.E, Standard Error; .*Chest complications at accident: including pneumothorax or subcutaneous emphysema, hemothorax, and pulmonary contusion; | | | | | | | | | | |
