# Supplementary material for: Impact of inflammatory biomarkers and surgical interventions on one-month recovery after rib fractures: A propensity-matched cohort study
Source: Surg Open Sci. 2025 Nov 3;28:49–62. doi: 10.1016/j.sopen.2025.10.009 (PMC12746880; doi:10.1016/j.sopen.2025.10.009)
Supplement: Supplementary Table 3 — Results of univariate and multivariable logistic regression analyses for NLR. [file mmc3.docx]

| Supply Table 3:Results of univariate and multivariable logistic regression analyses for NLR | | | | | | | | | | |
| --- | --- | --- | --- | --- | --- | --- | --- | --- | --- | --- |
| Variables | Univariate logistic regression analyses | | | | | Multivariable logistic regression analyses | | | | |
|  | Coef | S.E | t | P | 95% CI | Coef | S.E | t | P | 95% CI |
| Sex |  |  |  |  |  |  |  |  |  |  |
| Female | Ref |  |  |  |  |  |  |  |  |  |
| Male | 4.37 | 2.32 | 1.88 | 0.065 | -0.28 - 9.01 |  |  |  |  |  |
| Smoking |  |  |  |  |  |  |  |  |  |  |
| NO | Ref |  |  |  |  |  |  |  |  |  |
| YES | -2.93 | 1.85 | -1.58 | 0.119 | -6.63 - 0.77 |  |  |  |  |  |
| Comorbidities |  |  |  |  |  |  |  |  |  |  |
| NO | Ref |  |  |  |  |  |  |  |  |  |
| YES | 3.03 | 1.90 | 1.60 | 0.116 | -0.77 - 6.83 |  |  |  |  |  |
| The number of rib fractures | 0.18 | 0.33 | 0.54 | 0.591 | -0.48 - 0.84 |  |  |  |  |  |
| Rib fracture dislocation number | 0.44 | 0.31 | 1.44 | 0.155 | -0.17 - 1.06 |  |  |  |  |  |
| Paraspinal rib fractures |  |  |  |  |  |  |  |  |  |  |
| NO | Ref |  |  |  |  |  |  |  |  |  |
| YES | -1.07 | 1.98 | -0.54 | 0.592 | -5.04 - 2.90 |  |  |  |  |  |
| ISS |  |  |  |  |  |  |  |  |  |  |
| ≤16 | Ref |  |  |  |  | Ref |  |  |  |  |
| ＞16 | 4.86 | 1.44 | 3.36 | <0.001 | 2.01 - 7.11 | 3.50 | 1.45 | 2.40 | 0.017 | 0.62 - 6.37 |
| ＞25 | 4.72 | 1.93 | 1.63 | 0.440 | -1.07 - 5.31 | 2.35 | 1.37 | 1.71 | 0.739 | -0.41 - 5.11 |
| *Chest complications at acciden |  |  |  |  |  |  |  |  |  |  |
| No Complications | Ref |  |  |  |  | Ref |  |  |  |  |
| 1 Complications | 0.88 | 2.73 | 0.32 | 0.748 | -4.59 - 6.35 | -0.98 | 1.18 | -0.3 | 0.412 | -3.34 - 1.39 |
| Multiple Complications (≥2) | 5.09 | 2.22 | 2.30 | 0.025 | 0.65 - 9.53 | 1.09 | 0.98 | 1.12 | 0.270 | -0.88 - 3.07 |
| Analgesic |  |  |  |  |  |  |  |  |  |  |
| NO | Ref |  |  |  |  |  |  |  |  |  |
| YES | -0.55 | 2.09 | -0.27 | 0.792 | -4.74 - 3.63 |  |  |  |  |  |
| Payment type |  |  |  |  |  |  |  |  |  |  |
| Accident-related 3rd party claim | Ref |  |  |  |  |  |  |  |  |  |
| Insured | 3.38 | 1.97 | 1.72 | 0.091 | -0.56 - 7.33 |  |  |  |  |  |
| Cost | -0.00 | 0.00 | -0.20 | 0.845 | -0.00 - 0.00 |  |  |  |  |  |
| Age | 0.18 | 0.09 | 2.00 | 0.050 | 0.00 - 0.37 | -0.03 | 0.04 | -0.67 | 0.505 | -0.11 - 0.06 |
| BMI | -0.01 | 0.35 | -0.02 | 0.986 | -0.70 - 0.69 |  |  |  |  |  |
| ICU |  |  |  |  |  |  |  |  |  |  |
| NO | Ref |  |  |  |  | Ref |  |  |  |  |
| YES | 6.44 | 2.96 | 2.17 | 0.034 | 0.50 - 12.37 | 1.62 | 1.31 | 1.23 | 0.223 | -1.02 - 4.26 |
| Location |  |  |  |  |  |  |  |  |  |  |
| Unilateral | Ref |  |  |  |  |  |  |  |  |  |
| Bilateral | -2.08 | 2.30 | -0.90 | 0.370 | -6.67 - 2.52 |  |  |  |  |  |
| Number of Fixed Rib Fractures | 0.65 | 0.51 | 1.28 | 0.204 | -0.37 - 1.67 |  |  |  |  |  |
| Antibiotics |  |  |  |  |  |  |  |  |  |  |
| NO | Ref |  |  |  |  |  |  |  |  |  |
| YES | 0.69 | 2.59 | 0.27 | 0.790 | -4.49 - 5.87 |  |  |  |  |  |
| Intraoperative bleeding volume | 0.01 | 0.02 | 0.33 | 0.742 | -0.03 - 0.04 |  |  |  |  |  |
| Drainage volume | 0.00 | 0.00 | 0.50 | 0.623 | -0.00 - 0.00 |  |  |  |  |  |
| Drainage time | 0.60 | 0.35 | 1.69 | 0.097 | -0.11 - 1.31 |  |  |  |  |  |
| Operative time | 0.00 | 0.02 | 0.25 | 0.805 | -0.03 - 0.03 |  |  |  |  |  |
| Injury-to-Surgery Time |  |  |  |  |  |  |  |  |  |  |
| 1≤ | Ref |  |  |  |  |  |  |  |  |  |
| ＜7 | -3.60 | 2.92 | -1.24 | 0.222 | -9.44 - 2.24 |  |  |  |  |  |
| ≥7 | -3.00 | 3.39 | -0.88 | 0.380 | -9.79 - 3.79 |  |  |  |  |  |
| Postoperative Complications |  |  |  |  |  |  |  |  |  |  |
| NO | Ref |  |  |  |  |  |  |  |  |  |
| YES | -1.18 | 2.18 | -0.54 | 0.590 | -5.55 - 3.18 |  |  |  |  |  |
| Chest complications one month after surgery |  |  |  |  |  |  |  |  |  |  |
| NO | Ref |  |  |  |  |  |  |  |  |  |
| YES | -0.87 | 2.31 | -0.38 | 0.708 | -5.49 - 3.75 |  |  |  |  |  |
| Oral analgesic use at one-month follow-up |  |  |  |  |  |  |  |  |  |  |
| NO | Ref |  |  |  |  |  |  |  |  |  |
| YES | 2.93 | 2.10 | 1.39 | 0.169 | -1.28 - 7.13 |  |  |  |  |  |
| ALB | -0.21 | 0.18 | -1.13 | 0.264 | -0.57 - 0.16 |  |  |  |  |  |
| SII | 0.00 | 0.00 | 15.43 | <0.001 | 0.00 - 0.01 | 0.00 | 0.00 | 10.24 | <0.001 | 0.00 - 0.01 |
| LMR | -1.73 | 0.30 | -5.74 | <0.001 | -2.34 - -1.13 | -0.42 | 0.20 | -2.14 | 0.037 | -0.82 - -0.03 |
| PLR | 0.04 | 0.01 | 5.58 | <0.001 | 0.02 - 0.05 | 0.00 | 0.00 | 0.17 | 0.865 | -0.01 - 0.01 |
| HGB | -0.01 | 0.05 | -0.12 | 0.851 | -0.12 - 0.10 |  |  |  |  |  |
| Hospital day | -0.06 | 0.05 | -1.23 | 0.222 | -0.16 - 0.04 |  |  |  |  |  |
| ALB, Albumin; BMI, Body Mass Index; HGB, Hemoglobin; ISS, Injury Severity Score; ICU, Intensive Care Unit; CI, Confidence Interval; SII, Preoperative Systemic Immune Inflammation Indices; LMR, Lymphocyte-to-Monocyte Ratio; NLR, Meutrophil-to-Lymphocyte Ratio; PLR, Platelet-to-Lymphocyte Ratio; S.E, Standard Error; .*Chest complications at accident: including pneumothorax or subcutaneous emphysema, hemothorax, and pulmonary contusion; | | | | | | | | | | |
