# Supplementary material for: Impact of inflammatory biomarkers and surgical interventions on one-month recovery after rib fractures: A propensity-matched cohort study
Source: Surg Open Sci. 2025 Nov 3;28:49–62. doi: 10.1016/j.sopen.2025.10.009 (PMC12746880; doi:10.1016/j.sopen.2025.10.009)
Supplement: Supplementary Table 4 — Results of univariate and multivariable logistic regression analyses for LMR. [file mmc4.docx]

| Supply Table 4:Results of univariate and multivariable logistic regression analyses for LMR | | | | | | | | | | |  |
| --- | --- | --- | --- | --- | --- | --- | --- | --- | --- | --- | --- |
| Variables | Univariate logistic regression analyses | | | | | Multivariable logistic regression analyses | | | | |  |
|  | Coef | S.E | t | P | 95% CI | Coef | S.E | t | P | 95% CI |  |
| Sex |  |  |  |  |  |  |  |  |  |  | |
| Female | Ref |  |  |  |  | Ref |  |  |  |  | |
| Male | -1.80 | 0.80 | -2.27 | 0.027 | -3.39 - -0.21 | -0.56 | 0.59 | -0.95 | 0.345 | -1.75 - 0.62 | |
| Smoking |  |  |  |  |  |  |  |  |  |  | |
| NO | Ref |  |  |  |  |  |  |  |  |  | |
| YES | 0.9 | 0.64 | 1.40 | 0.168 | -0.39 - 2.19 |  |  |  |  |  | |
| Age | -0.03 | 0.03 | -0.94 | 0.350 | -0.1- 0.03 |  |  |  |  |  | |
| BMI | -0.03 | 0.12 | -0.28 | 0.782 | -0.27 - 0.21 |  |  |  |  |  | |
| Comorbidities |  |  |  |  |  |  |  |  |  |  | |
| NO | Ref |  |  |  |  | Ref |  |  |  |  | |
| YES | -1.54 | 0.64 | -2.40 | 0.02 | -2.82 - -0.25 | -0.53 | 0.48 | -1.11 | 0.272 | -1.49 - 0.43 | |
| The number of rib fractures | 0.05 | 0.11 | 0.47 | 0.643 | -0.18 - 0.28 |  |  |  |  |  | |
| Rib fracture dislocation number | 0.06 | 0.11 | 0.57 | 0.569 | -0.15 - 0.28 |  |  |  |  |  | |
| Location |  |  |  |  |  |  |  |  |  |  | |
| Unilateral | Ref |  |  |  |  |  |  |  |  |  | |
| Bilateral | 1.13 | 0.79 | 1.44 | 0.156 | -0.45 - 2.71 |  |  |  |  |  | |
| Paraspinal rib fractures |  |  |  |  |  |  |  |  |  |  | |
| YES | Ref |  |  |  |  |  |  |  |  |  | |
| NO | -0.16 | 0.69 | -0.23 | 0.82 | -1.54 - 1.22 |  |  |  |  |  | |
| ISS |  |  |  |  |  |  |  |  |  |  | |
| ≤16 | Ref |  |  |  |  |  |  |  |  |  | |
| ＞16 | -0.46 | 1.01 | -0.45 | 0.654 | -2.49 - 1.57 |  |  |  |  |  | |
| ＞25 | -0.21 | 1.09 | -0.20 | 0.846 | -2.39 - 1.96 |  |  |  |  |  | |
| *Chest complications at acciden |  |  |  |  |  |  |  |  |  |  | |
| No Complications | Ref |  |  |  |  |  |  |  |  |  | |
| 1 Complications | -0.38 | 1.00 | -0.38 | 0.707 | -2.37 - 1.62 |  |  |  |  |  | |
| Multiple Complications (≥2) | -0.72 | 0.81 | -0.90 | 0.374 | -2.34 - 0.90 |  |  |  |  |  | |
| Analgesic |  |  |  |  |  |  |  |  |  |  | |
| NO | Ref |  |  |  |  |  |  |  |  |  | |
| YES | 1.29 | 0.71 | 1.83 | 0.073 | -0.12 - 2.70 |  |  |  |  |  | |
| Payment method |  |  |  |  |  |  |  |  |  |  | |
| Self funded | Ref |  |  |  |  | Ref |  |  |  |  | |
| Medical insurance | -1.56 | 0.67 | -2.32 | 0.024 | -2.90 - -0.21 | -0.11 | 0.50 | -0.23 | 0.823 | -1.13 - 0.90 | |
| Cost | 0.00 | 0.00 | 1.64 | 0.106 | -0.00 - 0.00 |  |  |  |  |  | |
| ****Injury-to-Surgery Time**** |  |  |  |  |  |  |  |  |  |  | |
| 1≤ | Ref |  |  |  |  |  |  |  |  |  | |
| ＜7 | -0.22 | 1.02 | -0.22 | 0.830 | -2.27 - 1.83 |  |  |  |  |  | |
| ≥7 | -0.38 | 1.19 | -0.316 | 0.753 | -2.76 - 2.01 |  |  |  |  |  | |
| ALB | 0.06 | 0.06 | 0.91 | 0.368 | -0.07 - 0.19 |  |  |  |  |  | |
| SII | -0.00 | 0.00 | -5.34 | <0.001 | -0.00 -0.00 | -0.00 | 0.00 | -0.40 | 0.692 | 0.00 - 0.00 | |
| PLR | -0.01 | 0.00 | -4.93 | <0.001 | -0.02 - -0.01 | -0.01 | 0.00 | -2.93 | 0.005 | -0.01- -0.00 | |
| NLR | -0.21 | 0.04 | -5.740 | <0.001 | -0.28 - -0.14 | -0.08 | 0.08 | -1.02 | 0.314 | -0.23- 0.08 | |
| HGB | 0.00 | 0.02 | 0.04 | 0.969 | -0.04, 0.04 |  |  |  |  |  | |
| Hospital day | 0.05 | 0.02 | -3.21 | 0.002 | 0.02 - 0.08 | 0.05 | 0.01 | 3.17 | 0.003 | 0.02 - 0.07 | |
| Postoperative Complications |  |  |  |  |  |  |  |  |  |  | |
| NO | Ref |  |  |  |  |  |  |  |  |  | |
| YES | 0.48 | 0.76 | 0.64 | 0.524 | -1.03 - 2.00 |  |  |  |  |  | |
| Chest complications one month after injury |  |  |  |  |  |  |  |  |  |  | |
| NO | Ref |  |  |  |  |  |  |  |  |  | |
| YES | 0.46 | 0.80 | 0.58 | 0.565 | -1.14 - 2.07 |  |  |  |  |  | |
| Oral analgesic use at one-month follow-up |  |  |  |  |  |  |  |  |  |  | |
| NO | Ref |  |  |  |  |  |  |  |  |  | |
| YES | -1.56 | 0.71 | -2.19 | 0.033 | -2.98 - -0.13 |  |  |  |  |  | |
| ALB | 0.06 | 0.06 | 0.91 | 0.368 | -0.07 - 0.19 |  |  |  |  |  | |
| Drainage time | 0.15 | 0.12 | 1.19 | 0.240 | -0.10 - 0.40 |  |  |  |  |  | |
| Drainage volume | 0.00 | 0.00 | 1.14 | 0.260 | -0.00 - 0.00 |  |  |  |  |  | |
| Antibiotics |  |  |  |  |  |  |  |  |  |  | |
| NO | Ref |  |  |  |  |  |  |  |  |  | |
| YES | -0.01 | 0.90 | -0.01 | 0.991 | -1.81 - 1.79 |  |  |  |  |  | |
| Number of Fixed Rib Fractures | -0.14 | 0.18 | -0.78 | 0.441 | -0.50 - 0.22 |  |  |  |  |  | |
| Loacation |  |  |  |  |  |  |  |  |  |  | |
| Unilateral | Ref |  |  |  |  |  |  |  |  |  | |
| Bilateral | 1.13 | 0.79 | 1.44 | 0.156 | -0.45 - 2.71 |  |  |  |  |  | |
| Intraoperative bleeding volume | 0.00 | 0.01 | 0.10 | 0.920 | -0.01 - 0.01 |  |  |  |  |  | |
| ICU |  |  |  |  |  |  |  |  |  |  | |
| NO | Ref |  |  |  |  |  |  |  |  |  | |
| YES | -0.31 | 1.07 | -0.29 | 0.773 | -2.45 - 1.83 |  |  |  |  |  | |
| BMI | -0.03 | 0.12 | -0.28 | 0.782 | -0.27 - 0.21 |  |  |  |  |  | |
| Age | -0.03 | 0.03 | -0.94 | 0.350 | -0.10 - 0.03 |  |  |  |  |  | |
| ALB, Albumin; BMI, Body Mass Index; HGB, Hemoglobin; ISS, Injury Severity Score; ICU, Intensive Care Unit; CI, Confidence Interval; SII, Preoperative Systemic Immune Inflammation Indices; LMR, Lymphocyte-to-Monocyte Ratio; NLR, Meutrophil-to-Lymphocyte Ratio; PLR, Platelet-to-Lymphocyte Ratio; S.E, Standard Error; .*Chest complications at accident: including pneumothorax or subcutaneous emphysema, hemothorax, and pulmonary contusion; | | | | | | | | | | |  |
