# Supplementary material for: Impact of inflammatory biomarkers and surgical interventions on one-month recovery after rib fractures: A propensity-matched cohort study
Source: Surg Open Sci. 2025 Nov 3;28:49–62. doi: 10.1016/j.sopen.2025.10.009 (PMC12746880; doi:10.1016/j.sopen.2025.10.009)
Supplement: Supplementary Table 5 — Results of univariate and multivariable logistic regression analyses for PLR. [file mmc5.docx]

| Supply Table 5:Results of univariate and multivariable logistic regression analyses for PLR | | | | | | | | | | |
| --- | --- | --- | --- | --- | --- | --- | --- | --- | --- | --- |
| Variables | Univariate logistic regression analyses | | | | | Multivariable logistic regression analyses | | | | |
|  | Coef | S.E | t | P | 95% CI | Coef | S.E | t | P | 95% CI |
| Sex |  |  |  |  |  |  |  |  |  |  |
| Female | Ref |  |  |  |  |  |  |  |  |  |
| Male | 53.10 | 39.51 | 1.34 | 0.184 | -26.00 - 132.19 |  |  |  |  |  |
| Smoking |  |  |  |  |  |  |  |  |  |  |
| NO | Ref |  |  |  |  | Ref |  |  |  |  |
| YES | -76.89 | 30.04 | -2.56 | 0.013 | -137.02 - -16.76 | -32.51 | 25.31 | -1.29 | 0.204 | -83.27 - 18.24 |
| Intraoperative bleeding volume | -0.19 | 0.30 | -0.62 | 0.536 | -0.79 - 0.41 |  |  |  |  |  |
| Comorbidities |  |  |  |  |  |  |  |  |  |  |
| NO | Ref |  |  |  |  |  |  |  |  |  |
| YES | 56.42 | 31.70 | 1.78 | 0.080 | -7.03 - 119.87 |  |  |  |  |  |
| The number of rib fractures | -0.96 | 5.53 | -0.17 | 0.863 | -12.03 - 10.12 |  |  |  |  |  |
| Rib fracture dislocation number | 4.67 | 5.20 | 0.90 | 0.374 | -5.75 - 15.08 |  |  |  |  |  |
| Paraspinal rib fractures |  |  |  |  |  |  |  |  |  |  |
| YES | Ref |  |  |  |  |  |  |  |  |  |
| NO | -35.37 | 33.05 | -1.07 | 0.289 | -101.53 - 30.79 |  |  |  |  |  |
| ISS |  |  |  |  |  |  |  |  |  |  |
| ≤16 | Ref |  |  |  |  |  |  |  |  |  |
| ＞16 | 91.29 | 46.41 | 1.97 | 0.054 | -1.64 - 184.23 |  |  |  |  |  |
| ＞25 | 98.04 | 49.66 | 1.97 | 0.053 | -1.41 - 197.48 |  |  |  |  |  |
| *Chest complications at acciden |  |  |  |  |  |  |  |  |  |  |
| No Complications | Ref |  |  |  |  |  |  |  |  |  |
| 1 Complications | -13.90 | 47.70 | -0.29 | 0.772 | -109.42 - 81.63 |  |  |  |  |  |
| Multiple Complications (≥2) | 37.35 | 38.70 | 0.97 | 0.339 | -40.15 - 114.86 |  |  |  |  |  |
| Analgesic |  |  |  |  |  |  |  |  |  |  |
| NO | Ref |  |  |  |  |  |  |  |  |  |
| YES | -26.19 | 34.94 | -0.75 | 0.457 | -96.13 - 43.75 |  |  |  |  |  |
| Payment type |  |  |  |  |  |  |  |  |  |  |
| Self funded | Ref |  |  |  |  | Ref |  |  |  |  |
| Medical insurance | 69.05 | 32.64 | 2.12 | 0.039 | 3.70 - 134.39 | 23.37 | 27.46 | 0.85 | 0.399 | -31.72 - 78.45 |
| Cost | -0.00 | 0.00 | -1.28 | 0.204 | -0.00 - 0.00 |  |  |  |  |  |
| ALB | -5.19 | 3.02 | -1.72 | 0.091 | -11.24 - 0.86 |  |  |  |  |  |
| Age | 1.62 | 1.59 | 1.02 | 0.312 | -1.56 - 4.79 |  |  |  |  |  |
| BMI | -1.48 | 5.80 | -0.26 | 0.799 | -13.08 - 10.12 |  |  |  |  |  |
| ICU |  |  |  |  |  |  |  |  |  |  |
| NO | Ref |  |  |  |  |  |  |  |  |  |
| YES | 58.92 | 51.17 | 1.15 | 0.254 | -43.51 - 161.35 |  |  |  |  |  |
| Location |  |  |  |  |  |  |  |  |  |  |
| Unilateral | Ref |  |  |  |  |  |  |  |  |  |
| Bilateral | -24.94 | 38.68 | -0.65 | 0.522 | -102.36 - 52.48 |  |  |  |  |  |
| Number of Fixed Rib Fractures | -3.45 | 8.65 | -0.40 | 0.691 | -20.78 - 13.87 |  |  |  |  |  |
| Antibiotics |  |  |  |  |  |  |  |  |  |  |
| NO | Ref |  |  |  |  |  |  |  |  |  |
| YES | 7.17 | 43.47 | 0.17 | 0.870 | -79.84 - 94.19 |  |  |  |  |  |
| Drainage volume | -0.01 | 0.02 | -0.58 | 0.564 | -0.05 - 0.03 |  |  |  |  |  |
| Drainage time | 9.60 | 5.96 | 1.61 | 0.113 | -2.33 - 21.54 |  |  |  |  |  |
| Operative time | -0.19 | 0.26 | -0.73 | 0.470 | -0.71 - 0.33 |  |  |  |  |  |
| Injury-to-Surgery Time |  |  |  |  |  |  |  |  |  |  |
| 1≤ | Ref |  |  |  |  |  |  |  |  |  |
| ＜7 | -35.88 | 49.37 | -0.73 | 0.470 | -134.75 - 62.99 |  |  |  |  |  |
| ≥7 | -37.18 | 57.42 | -0.65 | 0.520 | -152.16 - 77.79 |  |  |  |  |  |
| **Postoperative Complications** |  |  |  |  |  |  |  |  |  |  |
| NO | Ref |  |  |  |  | Ref |  |  |  |  |
| YES | -80.00 | 35.17 | -2.27 | 0.027 | -150.41 - -9.59 | -57.92 | 28.79 | -2.01 | 0.049 | -115.67 - -0.17 |
| Chest complications one month after surgery |  |  |  |  |  |  |  |  |  |  |
| NO | Ref |  |  |  |  |  |  |  |  |  |
| YES | -32.26 | 38.58 | -0.84 | 0.407 | -109.49 - 44.98 |  |  |  |  |  |
| Oral analgesic use at one-month follow-up |  |  |  |  |  |  |  |  |  |  |
| NO | Ref |  |  |  |  |  |  |  |  |  |
| YES | -19.35 | 35.77 | -0.54 | 0.591 | -90.95 - 52.24 |  |  |  |  |  |
| SII | 0.05 | 0.01 | 5.31 | <0.001 | 0.03 - 0.07 | 0.01 | 0.02 | 0.64 | 0.525 | -0.03 - 0.06 |
| LMR | -26.29 | 5.33 | -4.93 | <0.001 | -36.96 - -15.62 | -11.54 | 6.10 | -1.89 | 0.064 | -23.77 - 0.69 |
| NLR | 9.92 | 1.78 | 5.58 | <0.001 | 6.36 - 13.48 | 4.23 | 3.92 | 1.08 | 0.286 | -3.64 - 12.09 |
| HGB | 0.80 | 0.92 | 0.87 | 0.387 | -1.04 - 2.63） |  |  |  |  |  |
| Hospital day | 0.57 | 0.84 | 0.69 | 0.495 | -1.10 - 2.25 |  |  |  |  |  |
| ALB, Albumin; BMI, Body Mass Index; HGB, Hemoglobin; ISS, Injury Severity Score; ICU, Intensive Care Unit; CI, Confidence Interval; SII, Preoperative Systemic Immune Inflammation Indices; LMR, Lymphocyte-to-Monocyte Ratio; NLR, Meutrophil-to-Lymphocyte Ratio; PLR, Platelet-to-Lymphocyte Ratio; S.E, Standard Error; *Chest complications at accident: including pneumothorax or subcutaneous emphysema, hemothorax, and pulmonary contusion; | | | | | | | | | | |
